# Tropism-Modification Strategies for Targeted Gene Delivery Using Adenoviral Vectors

**DOI:** 10.3390/v2102290

**Published:** 2010-10-13

**Authors:** Lynda Coughlan, Raul Alba, Alan L. Parker, Angela C. Bradshaw, Iain A. McNeish, Stuart A. Nicklin, Andrew H. Baker

**Affiliations:** 1 Institute of Cardiovascular and Medical Sciences, College of Medical, Veterinary and Life Sciences, University of Glasgow, 126 University Place, Glasgow G12 8TA, UK; E-Mails: Lynda.Coughlan@glasgow.ac.uk (L.C.), Raul.Alba@glasgow.ac.uk (R.A.), Alan.Parker@glasgow.ac.uk (A.L.P.); Angela.Bradshaw@glasgow.ac.uk (A.C.B.); Stuart.Nicklin@glasgow.ac.uk (S.A.N.); 2 Centre for Molecular Oncology and Imaging, Institute of Cancer, Barts and The London School of Medicine and Dentistry, Charterhouse Square, London EC1M 6BQ, UK; E-Mail: i.a.mcneish@qmul.ac.uk

**Keywords:** adenovirus, retargeting, detargeting, tropism, ligand, capsid protein

## Abstract

Achieving high efficiency, targeted gene delivery with adenoviral vectors is a long-standing goal in the field of clinical gene therapy. To achieve this, platform vectors must combine efficient retargeting strategies with detargeting modifications to ablate native receptor binding (*i.e.* CAR/integrins/heparan sulfate proteoglycans) and “bridging” interactions. “Bridging” interactions refer to coagulation factor binding, namely coagulation factor X (FX), which bridges hepatocyte transduction *in vivo* through engagement with surface expressed heparan sulfate proteoglycans (HSPGs). These interactions can contribute to the off-target sequestration of Ad5 in the liver and its characteristic dose-limiting hepatotoxicity, thereby significantly limiting the *in vivo* targeting efficiency and clinical potential of Ad5-based therapeutics. To date, various approaches to retargeting adenoviruses (Ad) have been described. These include genetic modification strategies to incorporate peptide ligands (within fiber knob domain, fiber shaft, penton base, pIX or hexon), pseudotyping of capsid proteins to include whole fiber substitutions or fiber knob chimeras, pseudotyping with non-human Ad species or with capsid proteins derived from other viral families, hexon hypervariable region (HVR) substitutions and adapter-based conjugation/crosslinking of scFv, growth factors or monoclonal antibodies directed against surface-expressed target antigens. In order to maximize retargeting, strategies which permit detargeting from undesirable interactions between the Ad capsid and components of the circulatory system (e.g. coagulation factors, erythrocytes, pre-existing neutralizing antibodies), can be employed simultaneously. Detargeting can be achieved by genetic ablation of native receptor-binding determinants, ablation of “bridging interactions” such as those which occur between the hexon of Ad5 and coagulation factor X (FX), or alternatively, through the use of polymer-coated “stealth” vectors which avoid these interactions. Simultaneous retargeting and detargeting can be achieved by combining multiple genetic and/or chemical modifications.

## 1. Introduction

Viruses are obligate intracellular parasites which have evolved as natural, biological delivery vehicles. This makes them an attractive choice of vector for various clinical gene therapy applications. Human adenoviruses (Ad) are currently the most widely used viral vectors for gene therapy for several reasons; their basic biology has been studied extensively, the viral genome can accommodate large heterologous transgene insertions, they readily infect quiescent and dividing cells, they can be amplified to high titers and they have previously been shown to be relatively safe for use in humans. The family *Adenoviridae* consists of five genera, including genus *Mastadenovirus* and genus *Aviadenovirus*, which infect mammals and birds respectively. The *Adenoviridae* are non-enveloped, icosahedral virions which contain a linear, monopartite, double-stranded DNA genome approximately 36 kb in size. As of now, there are at least 55 different human adenoviruses (species A–G, including subspecies B1/B2) which can be distinguished on the basis of their serological cross-reactivity, hemagglutinating properties or according to their phylogenetic sequence similarity (Table 1) [1–8]. Genomics, bioinformatics and restriction enzyme patterns were recently used to classify new human Ad (HAdV) species, HAdV-G52, HAdV-D53, HAdV-D54 and HAdV-B55 [9–11]. The adenoviral vector most commonly used for clinical trials and experimental gene therapy applications is species C adenovirus, HAdV-C5 (referred to as Ad5 in this review).

### 1.1. Adenovirus Structure

**Figure 1:**
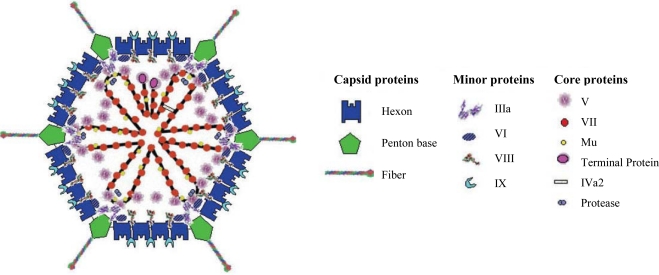
Adenovirus Structure. Schematic representation of the capsid and core proteins of an adenovirus. Figure reproduced with permission from Russell, W.C. Adenoviruses: update on structure and function. *J. Gen. Virol.* **2009**, *90*, 1–20 [15].

Adenoviruses contain 13 structural proteins (Figure 1), assigned with a numbering order from II–X, including, IIIa, Mu, TP, IVa2 [12], the protease which is putatively associated with interior of the icosahedron vertices [13] and L1-52/55K, which has been proposed to act as a scaffolding protein during viral assembly [14–16]. A nucleoprotein core complex surrounds the genome. This complex consists of a core-penton bridging protein (V), histone-like protein (VII), Mu protein and a Terminal Protein (TP) which is covalently attached to the 5□ end of the viral genome [17,18]. Together, adenoviral structural proteins are responsible for stabilization of the genome and encapsidation of the nucleoprotein core. The icosahedral capsid is composed of seven polypeptides; the trimeric hexon (II), which is complexed with three minor capsid polyproteins (VI, VIII and IX) which provide stabilization, the penton base (III), the penton-associated protein (IIIa) which bridges the hexon-penton base and the receptor binding fiber (IV) protein [19,20]. The fiber is composed of three domains; the tail at the N-terminus, the rod-like shaft and the globular knob domain at the C-terminus. The Ad5 fiber shaft consists of three intertwined strands made up of a number of β-repeats, each composed of 15 amino acids, with a putative heparan sulfate binding site, the KKTK motif [21–23]. The fiber exists as a glycosylated homotrimer, non-covalently complexed to the pentameric penton base protein (III) at the N-terminus [24]. This complex is also known as the penton capsomere. These trimeric complexes are embedded at the 12 vertices of the icosahedron structure, extending as protrusions on the external viral surface [25].

### 1.2. In Vitro Entry Pathway of Ad5

**Figure 2:**
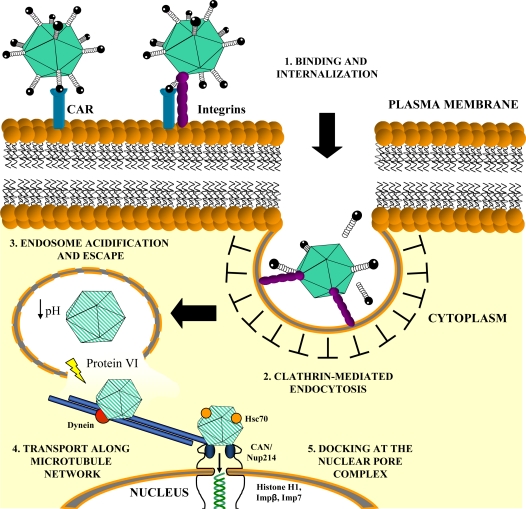
*In Vitro* Entry Pathway of Ad5. **1.** Ad5 attachment is mediated by binding of the fiber knob to the 46 kDa transmembrane receptor CAR [26–32]. **2.** An interaction between the RGD motif with the penton base triggers internalization by clathrin-mediated endocytosis, via ανβ3/5 integrins [33]. **3.** Partial disassembly of the capsid is induced upon acidification of the endosome [43]. Endosomal escape is modulated through the lytic action of protein VI [45]. **4.** The nucleocapsid-hexon core is translocated to the nuclear pore complex (NPC) along the microtubule network using the microtubule-associated motor, dynein [46,47]. **5.** The capsid undergoes its final dissociation event at the nuclear pore complex [47], allowing the core DNA to extrude into the nucleus for subsequent transcription and replication [48].

The two-step entry pathway of Ad5 *in vitro* (Figure 2) is initiated by a docking process in which the distal knob of the fiber binds to target cells via the 46 kDa, transmembrane coxsackie and adenovirus receptor (CAR) [26–32]. Fiber-CAR attachment is followed by the interaction of an arginine-glycine-aspartic acid (RGD) motif in the penton base with ανβ3/ανβ5 integrins, which subsequently triggers viral internalization [33]. It is thought that the Ad5 penton base-integrin interaction results in integrin clustering which activates signaling pathways, such as phosphoinositide-3-OH kinase (PI3K) [34,35], p38 mitogen-activated protein kinase (MAPK) [36,37] and extracellular signal-related kinase (ERK1/2)/p44/42 MAPK [37], inducing downstream effects which result in the polymerization and reorganization of actin filaments [35,38]. Recent data have shown that Ad5 binding to CAR leads to the activation of p44/42 MAPK, which promotes the dimerization and clustering of CAR, in addition to increasing the activation status of β1 and β3 integrin subunits [39]. Viral internalization is mediated via clathrin-mediated endocytosis [40,41], followed by partial capsid disassembly upon acidification of the endosome [42]. Endosomal escape is modulated by the lytic action of protein VI, after which the nucleocapsid is translocated to the perinuclear envelope along the microtubule network [38,43,44]. Transport to the nuclear pore complex involves the microtubule-dependent motor, cytoplasmic dynein, which facilitates Ad attachment to microtubules [45,46]. Capsid interactions with CAN/Nup214, recruit Hsc70 and nuclear histone H1 and H1 import factors, importin β and importin 7, which facilitate complete capsid disassembly and delivery of viral genomic DNA to the nucleus [48,49].

**Figure 3:**
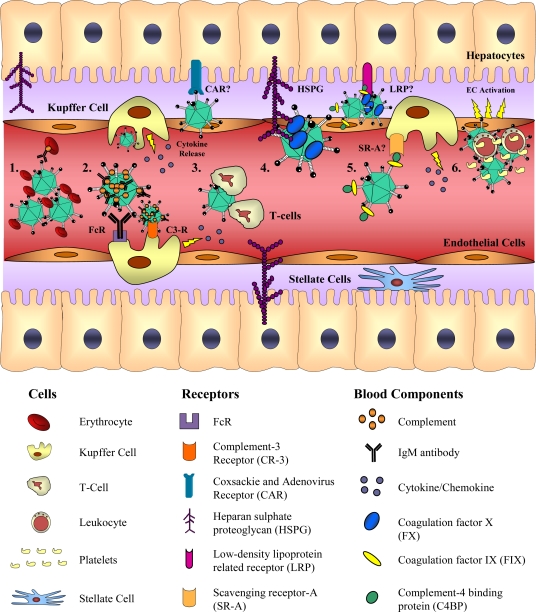
Reported Interactions of Ad5 with Blood Components *In Vivo*. **1.** Ad5 binding to CAR-expressing erythrocytes (species-specific expression of CAR) can cause trapping of virus in the circulation [81,82]. In the presence of antibody and complement, Ad5 can bind human erythrocytes via CR-1 [81]. **2.** Opsonization of Ad5 with natural IgM and/or complement promotes KC uptake via complement receptor-3 (CR-3) or Fc Receptor [83]. **3.** Ad interactions with T-cells [84]. **4.** FX binding to the Ad5 hexon promotes hepatocyte entry through HSPGs [66]. **5.** FIX/C4BP binding to the fiber knob has been proposed to mediate hepatocyte entry via HSPGs or LRP, and has been suggested to direct KC uptake [65]. **6.** Ad binding to platelets has been shown to enhance uptake by KCs [79]. Von Willebrand factor (vWF) and P-selectin have been associated with the formation of activated platelet-leukocyte aggregates which are cleared by scavenging macrophages [85].

It is now known that the *in vitro* process of infection by Ad5 can also involve alternative receptors and co-receptors to CAR and ανβ3/ανβ5 integrins. Heparan sulfate proteoglycans (HSPGs) have been shown to permit binding of Ad5 in the absence of CAR in A549 and CHO-K1 cells [49,50]. Additionally, vascular cell adhesion molecule 1 [51] and MHC class I [52] have been proposed to facilitate low affinity interactions with Ad5. However, Davison and colleagues concluded that Ad5 bound CAR, but not MHC class I allele HLA-A*0201, when these receptors were expressed on the surface of hamster cells [53]. Furthermore, McDonald and colleagues corroborated these data [54]. Additional integrins, ανβ1, α3β1, α5β1 and αMβ2 have also been shown to facilitate the internalization of adenoviruses *in vitro* [55–59]. More recently, a number of important interactions have been identified which have particular relevance *in vivo*, especially following intravenous (*iv*) delivery of Ad5 in mice (Figure 3). These will be discussed in the section below.

### 1.3. Bridging Receptors for Adenovirus Entry

Several groups have shown that direct interactions between the capsid of several adenoviral serotypes and various factors including complement-4 binding protein (C4BP), factor IX (FIX), VII (FVII), protein C, but predominantly factor X (FX), can mediate hepatocyte transduction via HSPGs [60–66]. Recently, the Ad5-FX complex has been shown to display a dependence on the sulfated side chains of liver HSPGs [67]. The authors demonstrated that removal of *N*-linked, but particularly *O*-linked sulfate side chains from HSPGs decreased FX-mediated infectivity enhancement *in vitro*. Furthermore, unlike native heparin, modified heparins lacking sulfation failed to inhibit the interaction of the Ad5-FX complex with the surface of hepatocytes following *iv* delivery. Interestingly, through the use of CAR-binding and/or penton base mutants, Bradshaw and colleagues also showed that trafficking of the Ad5-FX complex retained the interaction with cellular integrins as co-receptors for internalization. Coagulation factors have also been proposed to mediate a role in Ad delivery to tissues other than the liver. Human adenoviral serotypes Ad5 and Ad31 have been shown to use FX and FIX to infect human respiratory and ocular epithelial cells *in vitro* (even at 1/100th the physiological level found in human tear fluid, plasma and saliva), suggesting that the hexon:FX pathway may have evolved to promote natural adenoviral infections [68]. Another mechanism proposed to enhance Ad5 cell entry includes the use of lactoferrin, an antimicrobial molecule abundant in many bodily fluids [69]. Human lactoferrin in tear fluid has been shown to enhance Ad infection in human epithelial cells independently of CAR, via an unidentified receptor [69]. Furthermore, lactoferrin has also been associated with CAR-independent Ad5 transduction of human dendritic cells (DC), via the C-type lectin receptor, DC-specific intercellular adhesion molecule-3-grabbing non-integrin (DC-SIGN) [70].

Intravascular delivery of Ads induces acute inflammation, which is characterized by the activation of multiple innate immune effectors. In mice, the innate response to Ad5 is biphasic [71–73]. The first phase, induced independently of viral gene expression, peaks between one and six hours post-injection and is followed by a secondary peak five to seven days post-injection, when the response is directed primarily against transgene expression [71–73]. Kupffer cells (KCs), the resident macrophages of the liver, rapidly scavenge and eliminate Ad5-based vectors from the circulation in mice and rats [73–76] and this interaction contributes to the induction of pro-inflammatory cytokines and chemokines [65,77–80].

In order to study the contribution of resident macrophages to the biodistribution and immune response to Ad vectors, clodronate-encapsulated liposomes or gadolinium chloride can be used experimentally to deplete, or inactivate KCs [86,87]. This can also be achieved by saturation of KCs, by pre-dosing with high doses of Ad5 prior to intravascular delivery of the vector of interest [88]. The mechanisms of Ad5-uptake by KCs *in vivo* are poorly understood, although scavenging receptor-A (SR-A) has been proposed to be involved [78]. Opsonization of Ad5 with natural IgM and/or complement has also been proposed to direct KC uptake through complement receptor-3 (CR3) or Fc receptor (FcR) interactions [83]. Additionally, it has been shown that direct binding of the Ad5 particle to platelets results in the formation of platelet-leukocyte aggregates which are cleared by the reticuloendothelial system [79]. The formation of the Ad5-platelet-leukocyte complex was subsequently shown to be dependent on P-selectin and von Willebrand factor [85].

The release of pro-inflammatory effectors from activated KCs *in vivo* can increase vector-related toxicity, and contribute to the extensive liver pathology observed with Ad5 [73]. Activation of complement-3 (C3), in response to Ad5-mediated cell damage, is thought to contribute to the induction of acute thrombocytopenia [89], a well-reported transient side effect associated with *iv* delivery of Ad5 [90,91]. However, Ad5 interactions with other cell types encountered in the circulation, including neutrophils [92,93], monocytes [93] or erythrocytes [81,82] may also affect the success of vector delivery and/or the induction of inflammation. The *in vitro* transduction efficiency of Ad5 was shown to be impaired by >1000-fold when a suspension of virus and human blood cells was added to a monolayer of A549 cells, supporting the notion that blood cell interactions may also impair targeted delivery *in vivo* [93]. Interestingly, this effect was not observed when murine cells were used in a parallel experiment. It has recently been highlighted that the expression of CAR on human erythrocytes, but not murine erythrocytes, mediates Ad5-mediated agglutination [81,82]. Obviously, this finding has a bearing on the translational relevance of targeting efforts performed in murine models. In support of this, using an hCAR-transgenic mouse model (in which CAR is expressed on the surface of erythrocytes), Carlisle and colleagues demonstrated that interactions between Ad5 and CAR-expressing erythrocytes led to extended circulation times. However, transplantation of washed human erythrocytes into immunodeficient mice precluded efficient extravasation into tumor xenografts [81]. *In vivo*, hemodynamic responses to Ad5, mainly characterized by an acute reduction in blood pressure in murine models, have been causatively associated with the activation of hepatic endothelial cells by Ad-stimulated KCs [72,94]. Furthermore, the release of pro-inflammatory mediators from KCs function as chemoattractants for infiltrating neutrophils, which have been reported to interact with opsonized Ad5 via complement receptor-1 (CR-1) and FcR [92].

In conclusion, these multiple interactions not only contribute to the hepatocellular damage, toxicity and induction of potent inflammatory responses associated with Ad5, but they can also be attributed to limiting the efficacy of vector delivery to target tissues *in vivo*. Consequently, these interactions pose a major challenge to the clinical application of *iv* administered therapeutic adenoviruses.

## 2. Retargeting Adenoviral Vectors

There are two main strategies for improving the selectivity of targeted delivery *in vivo*, transcriptional targeting and transductional retargeting. Transcriptional selectivity limits transgene expression to target tissues and can be achieved by two means, genetic complementation [95,96] or through the use of tissue-specific promoters to drive viral replication [97–99]. Such adenoviral vectors are described as conditionally replicating adenoviruses (CRAds). Complementation strategies are dependent on an understanding of the underlying interactions between viral and cellular molecular networks. Such approaches are typified by the genetic modification or deletion of viral effectors whose functions are essential for productive infection in normal cells, but are redundant in target cells (e.g. malignant tissue). An example of a genetic complementation approach is the introduction of a Δ24 bp deletion (also known as *dl*922–947) within a conserved region (CR2) of the Ad5 early protein, the trans-acting transcriptional activator, E1A [96,100]. Binding of E1A to the tumor suppressor retinoblastoma protein, pRb, enables the virus to drive cell cycle progression, creating a cellular environment conducive to viral replication. Therefore, in normal cells, this Δ24 bp deletion effectively abolishes CR2-mediated binding to pRb and subsequently, viral replication. The design of such vectors exploits the knowledge that tumor cells frequently possess dysfunctional, or non-functional pRb tumor suppressor proteins [101]. Thus, the introduced viral defect results in the attenuation of viral replication in normal cells, but as the function of the defective viral protein is dispensable in tumor cells, replication is allowed to progress unaffected. The use of tissue-specific promoters is another widely used approach to refine the selectivity of Ad-delivered transgene expression. Such strategies can be applied to a wide range of tissue types and are not limited to malignant tissue. For example, inserted promoters have included the neuronal-specific neuron-specific enolase (NSE) promoter, the astrocyte-specific glial fibrillary acidic protein (GFAP) promoter [102], an alveolar epithelial type II cell-specific promoter, surfactant protein C (SP-C) promoter [103], and for transcriptional targeting in vascular tissue or endothelial cells, the intercellular adhesion molecule-2 (ICAM-2) or fms-like tyrosine kinase receptor-1 (FLT-1) promoters [104,105]. Achieving targeted transgene expression through the use of CRAds is a broad field and will therefore not be discussed further in this review article, as we will focus on transductional retargeting strategies.

The ultimate goal of viral gene therapy is to generate a non-toxic and self-localizing vector which is capable of high efficiency delivery to defined tissue types. The vast majority of these efforts employ strategies to redirect Ad5-infection to malignant tissue, including disseminated metastases. Since CAR is the primary receptor for Ad5 *in vitro*, it has long been considered to contribute to tumor transduction *in vivo*. However, several studies have reported the low expression of CAR in carcinoma lines, tumor explants and pathological specimens [106–111]. Furthermore, downregulation of CAR is thought to correlate with tumor progression and advanced disease states [108,109,112]. Thus, it is thought that low-level CAR expression may render tumor cells somewhat refractory to adenoviral infection *in vivo*, or at least impair intratumoral spread [113–115]. This, together with the finding that human erythrocytes express CAR on their surface, has highlighted the importance of developing CAR-independent retargeting strategies, as Ad5 agglutination of CAR-expressing erythrocytes has also been shown to limit efficient tumor delivery *in vivo* [81]. Applications which aim to treat metastatic disease favor *iv* delivery of Ad-based therapeutic agents. This necessitates the incorporation of retargeting modifications within viral structural proteins, to redirect the tropism of Ad-vectors to cancer-specific biomarkers. Moreover, these modifications should be combined with the introduction of detargeting mutations, which ablate native receptor and indirect bridging interactions. In summary, a truly retargeted vector should combine high efficiency retargeting and native receptor binding ablation, with strategies for the avoidance of the reticuloendothelial system and/or blood components. These vector features are key considerations for the development of optimal, *iv* administered Ad-based therapies.

**Figure 4:**
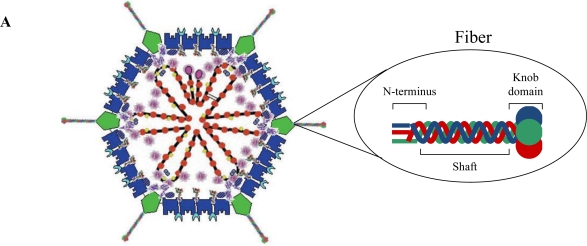

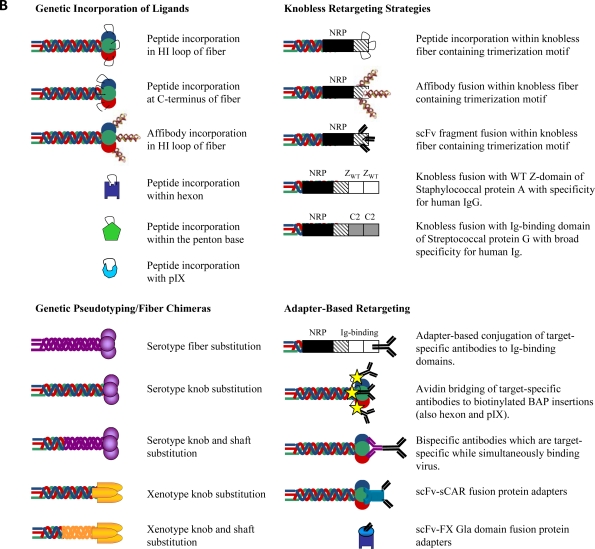
Retargeting Strategies for Adenoviral Vectors. **(A)** Schematic representation of the adenoviral capsid, highlighting the fiber region and its structural domains. Figure adapted with permission from Russell, W. (2009), *Journal of General Virology,* 90, 1–20, 2009 [13]. **(B)** Adenoviral retargeting strategies, genetic and adapter-based. Abbreviations are as follows; NRP = Neck region peptide from human lung surfactant protein D (to provide trimerization), ZWT = Wild type immunoglobulin (Ig)-binding region from the Z-domain of Staphylococcal protein A, C2 = Ig-binding domain from Streptococcal protein G, BAP = biotin acceptor peptide, scFv = single chain Fv antibody fragment, sCAR = soluble Coxsackie and Adenovirus Receptor and FX = factor X. Adapted with permission from Macmillian Publishers Ltd: *Oncogene*, Mathis *et al*., Oncolytic adenoviruses - selective retargeting to tumor cells. Nov 2005; 24:7775–7791. Copyright 2005 [123].

Various transductional retargeting strategies have been described which include the genetic incorporation of heterologous binding ligands to redirect vector tropism (*Section 2.1.*), capsid protein substitutions or “genetic pseudotyping” between divergent Ad serotypes, species or even a viral genus/family with differential tropism (*Section 2.2.*), or adapter conjugate strategies, based on the addition of an adapter molecule to crosslink the vector to a cellular target receptor (*Section 2.3.*). A schematic outline of these strategies is presented in Figure 4. Detargeting strategies have included; (i) genetic ablation of native tropism determinants (CAR/HSPGs/integrins), (ii) genetic ablation of “bridging” interactions and (iii) chemical shielding of capsid components using polymer-based strategies. Successful retargeting and detargeting has been achieved using combinations of genetic and/or chemical modifications.

### 2.1. Transductional Retargeting by Genetic Incorporation of Ligands

The genetic incorporation of retargeting ligands results in one-component therapeutic vehicles, which in the case of oncolytic vectors, can be propagated through multiple rounds of viral replication [123]. Ad5 capsid sites which can tolerate the genetic insertion of retargeting ligands include the C-terminus of the fiber [124,125], the HI loop of the fiber [126–128], the penton base [129,130], certain hypervariable regions of the hexon [131] and the minor capsid protein, pIX [125,132–135]. However, the success of these approaches depends on retention of the structural integrity of the selected capsid protein. The multimerization of viral structural proteins is often a prerequisite for efficient capsid assembly, and therefore insertions within these sites must not disrupt the molecular interactions required for adequate viral assembly [132,136]. Additionally, the heterologous ligand must retain its functional targeting capacity without the necessity for any major post-translational modifications. This is due to the nature of Ad protein translation and subsequent virion assembly; events which occur under non-reducing conditions in the cytosol and nucleus, respectively [137,138]. As a result, the genetic incorporation of many targeting ligands is limited by incompatibility between the inserted ligand and the Ad fiber. These incompatibilities can be due to alterations in the intracellular trafficking of the virus conferred by the ligand, which could result in degradation or endosomal recycling of the tropism-modified vector, and thus failure to reach the nucleus. Furthermore, ligands which require extensive post-translational modification within the endoplasmic reticulum (ER) are not suitable for genetic incorporation into adenoviral vectors, as the fiber protein does not enter the ER between its translation in the cytoplasm and its return to the nucleus for virion assembly.

#### 2.1.1. Fiber Retargeting Strategies: Peptide Incorporation

The distal, carboxy (C)-terminus of the fiber protein was first used for the genetic incorporation of the terminal decapeptide of gastrin releasing peptide [139]. Further studies followed with the incorporation of a heparin-binding polylysine motif (pK7 or K20) or an RGD motif [140–143]. These strategies were successful in enhancing the *in vitro* infection of a panel of CAR-deficient cell lines, including CF-KM4 and MM-39 cells (serous cell lines from submucosal tracheal glands) [140], glioma cells [143], endothelial and smooth muscle cells [142]. More recently, the 11 amino acid (aa) protein transduction domain of HIV-1 TAT was incorporated into the C-terminus of the Ad5 knob, resulting in improved CAR-independent transduction *in vitro* and enhanced tumor transduction *in vivo* [144]. However, due to disruptions to the trimerizing capacity of the fiber, attempts to incorporate C-terminal peptides >32 residues have largely been limited [142,145]. An exception to this was the successful incorporation of an 89 aa biotin acceptor peptide (BAP)-c-myc-linker fusion into the C-terminus, which retained fiber trimerization and was successfully biotinylated at this site [146].

The resolution of the crystal structure of the Ad5 fiber knob domain by X-ray crystallography identified the HI loop as a region suitable for peptide incorporation [147]. The rationale for this was that the HI loop was exposed on the surface of the fiber knob and therefore immediately accessible at the virion surface for receptor interactions; the HI loop had no involvement in the native tropism of Ad5, it was innately flexible and its length varied greatly between Ad serotypes [127]. The combination of these characteristics suggested that this domain could tolerate ligand insertion without disrupting the correct folding and trimerization of the knob domain. Initial proof-of-principle studies involved inserting non-targeting FLAG or hemagglutinin-(HA) epitopes into the HI loop in an attempt to demonstrate the suitability of this capsid location for peptide insertion [127,148]. To date, the HI loop has been shown to tolerate insertions of up to 83 aa with minimal detrimental effects on virion structural integrity or viral titer [126]. Reported insertions range from rationally selected motifs, such as the A20FMDV2 peptide, derived from Foot and Mouth Disease Virus (FMDV), which selectively targets ανβ6 integrin [149], the cysteine-constrained RGD-4C peptide (CDCRGDCFC) which targets RGD-binding integrins [125,128] or the YSA peptide, directed towards the Ephrin A2 receptor [150], to various candidate peptides screened using phage display technologies. Ligands identified using phage display libraries have proven useful for selecting markers which are potentially accessible on target tissues *in vivo* [151]. With this aim in mind, specific ligands identified by phage display and incorporated into Ad-vectors include an asparagine-glycine-arginine (NGR)-containing peptide directed towards Aminopeptidase N [152], a decapeptide GHPRQMSHVY ligand targeted to human tracheal glandular cells [153], ligands for human transferrin receptor [154,155], the endothelial-cell binding SIGYLPLP peptide [156], the linear EYHHYNK peptide which targets smooth muscle cells [157] and peptides with enhanced homing to the kidney [158]. In the latter study, Denby and colleagues demonstrated the *in vivo* targeting efficacy of an Ad-vector featuring renal targeted peptides, HTTHREP and HITSLLS, which were selected by phage display technology [158].

Many cancer retargeting strategies are designed to enhance delivery to malignant tissue or to target the endothelial networks which supply the tumors. Integrin-retargeting strategies using Ad5-RGD-4C, have resulted in RGD-dependent transduction enhancement of a wide range of carcinoma cell lines which express ανβ3/5 integrins [128,159–162], and increased the transgene expression profile following intravascular delivery [163]. Likewise, retargeting to HSPGs using polylysine-modified Ad5-pK7, enhanced CAR-independent transduction of myeloma, glioma, rhabdosarcoma and various other carcinoma cells *in vitro* and *in vivo* [164–168]. However, achieving tumor-specific delivery is still an important goal for cancer gene therapy. The disadvantage with the aforementioned retargeting strategies (RGD-4C and pK7) is that these ligands do not necessarily mediate cancer-selective transduction, as ανβ3/5 integrin and HSPG expression *in vivo* are not limited to malignant tissue [169–172]. Unfortunately, there appears to be a limited repertoire of identified and suitable, high affinity peptide ligands which could specifically target surface-expressed, tumor-specific biomarkers. Nonetheless, several attempts to achieve tumor-selective delivery have been documented. The epithelial-specific integrin, ανβ6, represents an attractive target for directed therapy since it is not expressed on normal adult epithelium, but is upregulated in numerous human carcinomas, including breast, lung, ovarian, cervical and colorectal, where it often correlates with poor prognosis [173–176]. Selective retargeting to ανβ6 integrin using a fiber-modified Ad5 vector featuring the RGDLXXL-containing A20FMDV2 peptide in the HI loop resulted in enhanced transduction *in vitro* which was independent of CAR and ανβ3/5 integrins [149]. This retargeting strategy resulted in a two-fold increase in viral delivery *in vivo,* using an ανβ6-overexpressing subcutaneous tumor xenograft model, which corresponded with improved tumor:liver genome ratios when compared to Ad5. However, the *in vitro* success of many tumor-specific retargeting strategies does not always translate *in vivo*. For example, despite dramatically improved transduction in human pancreatic carcinoma cells *in vitro*, retargeting of Ad5 to the Ephrin A2 (EphA2) receptor did not result in increased adenoviral targeting to pancreatic, subcutaneous xenografts [150]. To overcome these limitations, it may be useful to generate vectors which feature additive retargeting insertions. Previous strategies featuring the dual incorporation of peptides (e.g. RGD-4C and pK7), within the C-terminus in conjunction with the HI loop, also demonstrated enhanced infectivity in both CAR-ve and CAR+ve cell lines [168,177]. These efforts may be improved upon by combining the presentation of high affinity, tumor specific ligands in various capsid configurations, including the C-terminus and HI loop of the fiber in conjunction with compatible hexon regions and/or optimized pIX insertion sites (see later).

Previously, the incorporation of fiber-compatible targeting ligands has been restricted to small peptide ligands which are capable of retaining their targeting function within the structural constraints exerted by the fiber [126,145,178]. However, several recent advances in vector engineering have now permitted the genetic incorporation of fusion proteins with increased complexity, as exemplified by Affibody molecules [179]. Affibodies are engineered, artificial protein ligands arranged in a three-α-helical bundle scaffold molecule structure [180]. To date, the Affibody protein framework chosen for the modification of adenoviral tropism has been based on the Z-domain derived from Staphylococcal protein A [137,179,181,182]. Affibodies with specific target-binding sites can be generated by simultaneous randomization of 13 amino acids on the Fc-binding face of the protein framework, creating libraries from which *in vitro* selection methods such as phage display can be used to identify candidate targeting moieties [183]. A recent study has demonstrated successful *in vitro* targeting to human epidermal growth factor receptor-2 (HER2)/neu, an important tumor antigen which is overexpressed in ovarian and breast carcinomas [184], through the incorporation of a HER2/neu-directed Affibody within the HI loop of a CAR-binding ablated fiber [179]. The optimal configuration for the inserted Affibody was found to be a dimeric tandem-repeat with flexible flanking sequences. This arrangement was thought to allow optimal domain folding of both the insertion and the neighboring fiber knob regions, and to increase the avidity of the Affibody for HER2/neu. Following these findings, Myhre and colleagues engineered an Ad5 vector with dual specificity, featuring both the HER2/neu-binding, linker-optimized Affibody and another Affibody molecule (*Taq*-polymerase binding) at different positions relative to each other within the HI loop of the fiber [182]. This approach may prove useful in combining high affinity retargeting strategies, which have the potential to be further enhanced by peptide display at alternative capsid sites.

#### 2.1.2. Fiber Retargeting Strategies: Generation of Knobless Fiber Shaft Fusions

One of the most intriguing strategies for retargeting in recent years has exploited advancements in the vector engineering of knobless Ad particles, which feature fiber shaft fusion proteins. This approach permits the genetic fusion of retargeting molecules into vectors which lack the native receptor binding domain contained within the fiber knob. However, truncation or complete removal of the fiber can lead to disruptions in the intramolecular interactions required for efficient viral entry, assembly and propagation, therefore affecting vector titers, infectivity and growth characteristics [185–188]. To compensate for the loss of fiber trimer formation, vectors have been genetically modified to contain foreign trimerization motifs, including those derived from Moloney murine leukemia virus (MoMuLV) [189], the reoviral σ1 protein [190], the fibritin motif from T4 bacteriophage [136,191] or the neck region peptide (NRP) from human lung surfactant protein D [187]. Using this approach, various modified Ad-vectors have successfully been used as platforms for genetic fusion-retargeting, for example by utilizing peptides, more complex protein ligands, Affibody targeting molecules or cytosol stable single-chain variable (scFv) fragments.

Initial studies involved generating knobless Ad vectors with a truncated fiber shaft which was replaced by a fibritin [136], MoMuLV [189] or reoviral σ1 protein trimerization motif [190], fused to a C-terminal, polyhistidine peptide ligand. These vectors were shown to mediate receptor-specific transduction *in vitro*, through an interaction with a surface expressed anti-His antibody [136,189]. Furthermore, the latter strategy, when combined with modifications in the penton-base which ablated integrin binding, resulted in a vector which displayed reduced hepatic tropism and enhanced bioavailability in mice [190]. More importantly, further efforts using the physiologically relevant targeting ligand RGD-4C, fused to the NRP trimerization signal, also demonstrated that the fusion vector was capable of RGD-dependent enhancement through integrins expressed on human carcinoma cell lines [187]. Using a more radical approach, Belousova and colleagues expanded this technology to incorporate the CD40 ligand (CD40L), a significantly larger and more structurally complex molecule than the small peptides tested in previous efforts [192]. Encouragingly, the CD40L-fusion retained its functional efficacy within the fiber chimera and succeeded in enhancing the infection of CD40-expressing carcinoma cells and human dendritic cells. Importantly, targeting via CD40L was shown to be successful *in vivo*, directing *iv* administered Ad to CD40-expressing pulmonary vasculature when using a hCAR transgenic mouse model [193]. In this study, the authors first delivered an adenoviral vector expressing hCD40 under the control of the FLT-1 promoter. Once selective expression of hCD40 in the pulmonary vasculature *in vivo* had been validated, animals were administered with the CD40-targeted vector, Ad5Luc.FF/CD40L, which featured the fibritin trimerization motif fused to the CD40 ligand, CD40L [193]. Retargeting to HER2/neu has also been achieved using a knobless, Affibody-based fibritin-fusion strategy [137]. These chimeric fibers were compatible with virion assembly and the resultant vectors successfully mediated transduction to HER2/neu-expressing cancer cells. However, the ability of this vector to selectively target the HER2/neu receptor *in vivo* has not yet been assessed.

Recently, an additional endogenous trimerization element was identified (although its precise sequence was not elucidated) within the N-terminus of the fiber shaft [194]. In this study, the authors’ generated truncated fiber constructs corresponding to the N-terminal tail with the first 6.5, 7, 7.5 and 9 shaft repeats, all of which were found to retain the ability to form stable homotrimeric fibers independently of the C-terminal knob. This finding was successfully exploited to support the fusion of a 240 aa scFv to carcinoembryonic antigen (CEA) or the 70 aa peptide, human insulin-like growth factor (IGF-1) to the truncated fiber shaft without the requirement for foreign trimerization motifs [194]. Both targeting moieties were shown to be capable of mediating receptor-dependent transduction enhancement *in vitro*, through CEA expressed on colorectal carcinoma cells, or the IGF-1 receptor on NIH 3T3 cells, respectively [194].

However, although these engineered vectors are promising in terms of their retargeting potential, viruses which lack the fiber knob domain often exhibit low yields and contain less fiber copies per virion than their unmodified counterparts. This may reflect the proposed auxiliary roles of the fiber knob in the virus life cycle, which includes mediating or contributing to fiber synthesis and encapsidation [195], virion assembly [196], virion maturation [185] and cell-cell spread during virus propagation [197]. In an effort to overcome these limitations, Hong and colleagues developed novel fiber chimeras (which retained the knob domain) consisting of a heterologous trimerization motif (NRP) fused to a targeting ligand, flanked on both sides by a linker sequence and featuring an activated FX (FXa) cleavage site upstream of the knob domain [153,198]. The retention of the knob domain during virus amplification and propagation facilitated high titer yields and efficient fiber encapsidation. Following viral production, the presence of the FXa cleavage site permitted proteolytic removal of the knob domain, thus exposing the inserted targeting ligand. Using this approach, inserted ligands included a decapeptide (GHPRQMSHVY) targeted to cystic fibrosis transmembrane regulator (CFTR)-deficient human tracheal glandular cells [153], the RGD tripeptide or an Affibody oligopeptide with specificity for the human IgG_1_ Fc domain [198]. Interestingly, the retargeting capacity of the Affibody insertion was retained without the proteolytic removal of the knob domain, suggesting that the Affibody ligand was accessible for interaction with its target receptor even within the fiber shaft domain [198]. In support of this, a recent study described the direct modification of a lysine-lysine-threonine-lysine (KKTK) motif within the fiber shaft to RGDK, which resulted in improved tumor cell infectivity and targeting *in vivo* [199]. Therefore, in the future it may be possible that the fiber shaft itself has the potential for combinatory retargeting strategies, either when applied with fiber knob alone and/or other capsid retargeting modifications. The genetic fusion strategies described above have also been designed for compatibility with adapter-based retargeting, which we will discuss later.

#### 2.1.3. Hexon Retargeting Strategies

The trimeric hexon is the most abundant capsid protein in the Ad virion, making this region an attractive option for maximizing heterologous ligand presentation. The identification of a number of hypervariable regions (HVRs) within the solvent-exposed loops on the hexon surface were highlighted as an alternative capsid site for the genetic incorporation of peptides [1,131,200,201]. This was supported by the fact that these regions were predicted to possess innate flexibility, an assumption based on their lack of discernible structure in early crystallographic studies [200].

The earliest attempt to modify the surface of the hexon involved introducing an 8 aa peptide from the VP1 capsid protein of poliovirus, resulting in a chimeric vector capable of inducing VP1-specific neutralizing antibodies [202]. Subsequently, HVR2 and HVR5 were identified as domains thought to be suitable for the optimal surface exposure of inserted epitopes [131,203]. In particular, HVR5 was thought to represent the most promising site for modification, due to the lack of residues involved in intramolecular interactions and the fact that it varied in length between different Ad species [200,201]. Studies using the neutralizing epitope DNPASTTNKDK from poliovirus as a model peptide, established an optimal linker-peptide configuration for the subsequent insertion of the αν-integrin specific ligand, GSDCRGDCFGS, into HVR5 [201]. Ad5 modified in HVR5 with GSDCRGDCFGS facilitated knob-independent entry via αν-integrins in HEK293 cells and enhanced the transduction of vascular smooth muscle cells, demonstrating that hexon retargeting strategies can circumvent the dependence on the CAR-entry pathway of Ad5. Similar studies demonstrated that HVR2 and HVR5 could tolerate the insertion of a 6-His peptide without detrimentally affecting capsid assembly, or the native Ad5 infectivity pathway, and that these inserted epitopes were exposed and readily detectable using an anti-His antibody [131]. Encouragingly, it was later shown that HVR5 could accommodate a 36 aa peptide without adversely affecting virus infectivity, growth, or stability [203]. Campos and Barry significantly improved on this by successfully introducing the 71-residue BAP protein at the same site (HVR5), again with minimal detrimental effects on virion assembly [204].

However, in a separate study, Kurachi and colleagues found that the genetic inclusion of an RGD motif within the hexon showed no effect in terms of retargeting [125]. This was contrary to the findings of Vigant and colleagues [201], but may be due to subtle differences in the amino acid sequence of the inserted RGD peptides (SRGSCDCRGDCFCGSPR in the former study and GSDCRGDCFGS in the latter study). In summary, despite the capacity to tolerate reasonably large heterologous insertions, the efficiency of hexon retargeting is variable. This may be a result of incompatibilities between the chosen ligand and virion assembly. Alternatively, it is possible that the structural conformation of the ligand may be restrained, therefore rendering it inaccessible within the trimeric hexon. It has also been proposed that the fiber could potentially mediate steric hindrance, impairing the interaction of hexon inserted ligands with their cognate target receptors [125]. With regard to this suggestion, it was later shown that hexon-retargeted, fiberless Ad-particles also failed to demonstrate effective retargeting [205]. Therefore, it appears that the success of retargeting strategies involving the insertion of peptides into the hexon may primarily be dependent on the choice of candidate ligand, and each effort may require ligand specific optimization.

Interestingly, Vigant and colleagues observed a reduction in liver gene transfer when comparing HVR5-modified vectors featuring the insertion of the RGD motif, or an 8 aa or 24 aa non-targeted peptide sequence composed of GA-repeat residues [64]. Consequently, in light of the recent discovery that a hexon-FX interaction mediates significant hepatocyte transduction [61,66], the insertion of heterologous peptides into the hexon could be optimized in the future and used in an attempt to impair binding of FX to the hexon. This could conveniently reduce the characteristic hepatotropism of Ad5 *in vivo*, while simultaneously attempting to retarget the vector. Additionally, as the Ad5 hexon is considered to be a major antigenic determinant for neutralizing antibody responses [206], insertions within the HVRs may result in the occlusion of antigenic target sites, permitting partial escape from neutralization while simultaneously achieving retargeting. This is particularly important as the high seroprevalence of pre-existing neutralizing antibodies (NAbs) to Ad5 in humans can limit its efficacy in clinical applications; resulting in rapid vector/transgene elimination or over-stimulation of inflammation through Fc-FcR interactions with immune cells [207,208].

#### 2.1.4. Alternative Capsid Retargeting Strategies: Penton Base and pIX

Alternative adenoviral capsid proteins, including the penton base and minor capsid protein pIX, have also been assessed for their suitability for ligand insertion. The penton base exists as a homopentameric protein, the monomer of which is ∼470–570 residues in length [209]. The penton base assembles into a non-covalent complex, the penton capsomer, with the homotrimeric fiber. The presence of an exposed RGD motif within the hypervariable loop region of the penton base of various Ad species, facilitates the engagement of cell surface integrins and it has also been suggested that multiple integrin receptors (up to five) bind each penton base, promoting integrin clustering and triggering efficient virion internalization [210]. Wickham and colleagues first described successful *in vitro* retargeting of recombinant penton base protein *in vitro*, by substitution of the RGD site for an LDV-containing peptide motif, which mediated binding to α4β1 integrin [211]. A subsequent study, demonstrated that HA, incorporated into the penton base of the virion, was capable of interacting with a membrane anchored, anti-HA scFv surrogate receptor on the surface of cells [130]. In a separate study, the insertion of a FLAG epitope into the Ad-penton base allowed adapter-based retargeting using a bispecific, anti-FLAG monoclonal antibody targeted to αν-integrins expressed on human endothelial and smooth muscle cells [129].

Protein pIX, is a minor capsid polypeptide of ∼140 aa, which is incorporated into the mature viral capsid and associates with hexon proteins on each facet of the icosahedral virion [212,213]. pIX is responsible for stabilizing hexon-hexon interactions [214], full length viral genome packaging [215] and has also been proposed to play a role in the nuclear reorganization and transcriptional activity of Ad5 [216]. However, the latter effect was observed under experimental conditions of transient pIX expression, and was subsequently shown to have little influence on the activation of Ad promoters during wildtype replication [217]. There are 240 copies of the pIX per virion, and these have been shown to be organized as four trimers per group-of-nine (GON) hexons [25,212]. The N-terminus of pIX is thought to be positioned at the middle of each facet. The C-terminus of pIX has been proposed to be surface exposed, forming a four-helix bundle arrangement, with one helix associated externally between hexons H2 and H4 of adjacent facets [218,219]. In agreement with its putative surface localization, inserted ligands at the C-terminus have been shown to be accessible for cellular receptor binding.

Dmitriev and colleagues successfully engineered a FLAG octapeptide and a polylysine motif into this region [220]. Both insertions were accessible for binding, and the polylysine motif successfully enhanced the infection of CAR-negative carcinoma cell lines in a knob-independent manner. Subsequent studies improved on this design by adding α-helical spacers to extend and improve ligand presentation from the carboxy-terminus of pIX [221]. Using this strategy, the authors efficiently presented a MYC-tag and the RGD motif, both of which were accessible for binding, such that the RGD insertion resulted in improved transduction in endothelioma cells, which lack the native receptors for Ad5. Furthermore, the insertion of the 71 aa BAP protein was also found to be optimal, in terms of detectable surface biotins, when it featured a 45Å α-helical spacer between pIX and BAP [222]. However, this pIX-modification strategy was not suitable for retargeting when biotinylated antibodies directed against CD59 and CD71 were conjugated to BAP. Encouragingly, the C-terminus of pIX has been shown to tolerate large proteins, including green fluorescent protein (GFP) [135], enhanced green fluorescent protein (EGFP) [133] and thymidine kinase (TK) from herpes simplex type-1 (HSV-1) [223]. Although it appears that the type of inserted ligand affects the efficiency of its presentation at the C-terminus of pIX, this site still represents an attractive site for the incorporation of heterologous ligands. This is due in part to the finding that trimerization of pIX is dispensable for both its inclusion in assembling virions and capsid stability [224].

### 2.2. Transductional Retargeting by Genetic Pseudotyping

Of the 55 distinct human adenoviruses, many exhibit differential tropism, mediated primarily by the interaction of the fiber protein with different cell surface receptors [188]. Pseudotype switching of adenoviral fiber proteins therefore represents a logical approach to transductional retargeting, allowing the alteration of viral tropism. The high fidelity of structural integrity, and the conserved homology of fiber tail domains amongst diverse Ad species, permits genetic engineering with minimal disruptions to the trimeric fiber [225,226]. Whole fiber replacement strategies have mostly focused on the substitution of the Ad5 fiber with fibers derived from species B adenoviruses [227], for which reported receptors include CD46 [117,228,229], in addition to CD80, CD86 [118], HSPGs [230], receptor “X” [119,229], or as yet unidentified receptors [231]. Fiber pseudotyped vectors Ad5/16, Ad5/11 and Ad5/35 were shown to improve the infection of human smooth muscle cells *in vitro* [232]. Similarly, efficient retargeting to CD34+ human hematopoietic stem cells has been achieved *in vitro* using Ad5 pseudotyped with the short shafted fiber of Ad35 [233]. Ad5/F35 expressing a GFP-tagged CFTR transgene, displayed superior transduction to Ad5-GFP-CFTR in cystic fibrosis (CF) and non-CF human airway epithelial cells and restored chloride channel function [234]. The localization of CAR on the basolateral surface of airway epithelia restricts efficient delivery of Ad5-based vectors to these cells for gene therapy applications [197,235]. However, the Ad5/F35-GFP-CFTR pseudotyped vector successfully entered *ex vivo* reconstituted human airway epithelia through the apical pole [234]. This is consistent with the localization of CD46 expression, which is found on the apical surface of normal human airway epithelia [236].

The overexpression of CD46 in many human cancers has prompted the investigation of Ad5/11 and Ad5/35-based vectors for potential tumor targeting applications [237–240]. Approaches using Ad5/35 have had varied tumor targeting efficacy *in vivo*, with reports of low level transduction of breast and liver metastases [237,239,241]. However, delivery of Ad5/35 to liver metastases was improved using a snake venom FX-binding protein (X-bp), to inhibit the Ad5 association with coagulation FX [237]. This strategy translated to improved antitumoral efficacy when using a therapeutic derivative of the vector, Ad5/35.IR-E1A/TRAIL, which expressed tumor necrosis factor-related apoptosis inducing ligand (TRAIL) transgene [237]. Interestingly, Wang and colleagues developed two Ad5/35-based vectors (Ad5/35+ and Ad5/35++) which featured higher affinity binding to CD46 [240]. The authors’ first generated mutant Ad35 knob proteins by mutagenic PCR, which were expressed in *E. coli*, purified and tested for their affinity for sCD46 binding. The corresponding sequences (N217D, T245P, I256L for Ad5/35+ and D207G and T245A for Ad5/35++), which conferred ∼3-fold and ∼23.2-fold higher affinities for CD46 respectively, were then incorporated into an Ad5/35 chimeric vector in an effort to improve its targeting capacity [240]. The purified Ad particles displayed ∼4-fold and ∼60-increased affinities for sCD46, respectively, as determined by Surface Plasmon Resonance (SPR). Although, this strategy did not result in dramatically improved delivery *in vitro* when compared to unmodified Ad5/35, transduction of CD46^high^ liver metastases was markedly increased following intravascular delivery with Ad5/35++. More recently, Alba and colleagues have demonstrated the benefit of pseudotyping a FX-binding ablated Ad5 vector with the high affinity fiber 35++ [243]. The resultant vector (Ad5CMVlacZ-HVR5*7*E451Q) mediated a significant improvement in lung:liver ratios when delivered intravascularly in macrophage-depleted CD46-transgenic mice [242].

Pseudotyped vectors based on Ad5/3 have demonstrated enhanced gene transfer to a broad range of cell types, including renal [243] and ovarian carcinoma [244], malignant glioma [245], melanoma [246] and Epstein Barr virus (EBV)-transformed B-lymphocytes [247]. More importantly, *in vivo* delivery of oncolytic Ad5/3 vectors prolonged the survival of mice with orthotopic human ovarian adenocarcinomas [248], subcutaneous and peritoneal metastatic renal carcinomas [249], intracranial glioma xenografts [250] and hormone-refractory prostate metastases [251]. Ad5/11 pseudotyped vectors have also augmented the transduction efficiency of a broad panel of human carcinoma lines when compared with Ad5 [228]. Furthermore, in a murine and non-human primate animal model system, both Ad5/35 and Ad5/11 pseudotyped vectors were shown to have an improved safety profile *in vivo*, with reduced toxicity and limited induction of inflammatory cytokines when compared to Ad5 [252].

Vectors pseudotyped with fibers from species D adenoviruses, including Ad17, Ad19, Ad24, Ad30, Ad33, Ad37, Ad43 and Ad47, which use CD46 [116], sialic acid [120,121], an unidentified receptor on the surface of murine dendritic cells [253] and/or αν integrins as receptors [122,254], often in addition to CAR [28], are also currently undergoing investigation for various applications. Interestingly, Ad5 vectors pseudotyped with fibers from Ad19 or Ad37 (Ad5/19p and Ad5/37), were reported to have reduced hepatic tropism following intravascular delivery in rats [255], highlighting their potential for use as platform vectors for retargeting. With this in mind, novel retargeting approaches have been designed to capitalize on the reduced infectivity often observed with rare Ad serotypes, such as Ad19p [158,256] and Ad41 [257]. The most promising retargeting advances have been made with pseudotype Ad5/19p, in which candidate peptides (HTTHREP and HITSLLS), identified by *in vivo* phage display, were incorporated into the HI loop of the Ad19p knob domain [158]. Intravenous delivery of HTTHREP and HITSLLS-targeted Ad5/19p vectors into rats resulted in selective transduction of renal tubular epithelium and glomeruli, respectively [158]. Furthermore, intravascular and intraperitoneal delivery of the HITSLLS-retargeted Ad5/19p resulted in comparable transduction of subcutaneous and peritoneal renal tumor xenografts to Ad5, which was accompanied with reduced liver transduction [256]. In a separate study, the exposed loop regions within the fiber of serotype Ad41 were assessed for their suitability for peptide incorporation [257]. Using the RGD-4C peptide as a model ligand, the authors demonstrated that ligand incorporation was tolerated within the EG, HI and IJ loop domains, as well as the C-terminus, with negligible effects on fiber trimerization. Ad5 vectors pseudotyped with these modified Ad41 fibers improved the *in vitro* transduction efficiency of various cell types, with the HI loop insertion displaying the best overall improvement. Novel “xenotyping” strategies involve the substitution of Ad5 knob proteins with those of non-human adenoviruses such as canine adenovirus (CAV-1 and CAV-2) [258] or members of the genus *Atadenovirus*, ovine atadenovirus type 7 (OAdV7) [260] and bovine atadenovirus [260]. Additionally, a successful fiber mosaic virus has been constructed by incorporating the trimeric σ1 spike protein from Reovirus into Ad5 [261,262]. This approach was made technically possible by the high degree of structural similarity between the receptor-binding determinants of these two distinct viral families. The generation of this mosaic virus resulted in CAR-independent transduction enhancement conferred by reoviral tropism determinants, junction adhesion molecule (JAM-1) and sialic acid [263,264].

Taken together, it is clear that detailed investigation of the tropism, biodistribution and toxicity profiles of diverse species of Ads may uncover serotypes with desirable *in vivo* characteristics, which may help to overcome the current limitations associated with Ad5. Therefore, improving our understanding of the tropism determinants of alternative Ads species will likely prompt the development of novel vector systems and expand the use of fiber pseudotyped viruses in the future. However, it is also worth considering that genetic pseudotyping has been shown to alter the intracellular trafficking of Ads, and can often result in reduced transduction as a result of inefficient endosomal escape (e.g. Ad7) [265], retarded nuclear translocation (e.g. Ad5/7), or retention of virus in late endosomes or lysosomes (e.g. Ad5/35) [186,188]. This may impact upon their use for gene therapy applications.

### 2.3. Transductional Retargeting by Conjugation of Ligands: Adapter Ligand Complexes

Adapter based transductional retargeting is achieved by cross-linking extraneous targeting entities to the virus, either by covalent or non-covalent interactions [123,225,266]. Additionally, multiple conjugate-based strategies can be combined to create multi-component targeting systems [182]. Adapters can consist of conjugated Ab fragments [267], bispecific adapters or anti-Ad diabodies [268–270] and recombinant adapter-fusion proteins [271–274]. Bispecific antibodies contain two distinct binding specificities and can exist in a number of formats, including tandem scFv-scFv, Fab conjugates and diabody single chain or tandem conformations [275,276]. Conveniently, with regard to adenoviral engineering, many of these approaches can be designed to retarget, while simultaneously detargeting from native receptor binding.

The conjugation of tissue-selective antibodies to adenoviral vectors has been achieved by genetic incorporation of an immunoglobulin (Ig) Fc-binding domain from staphylococcal protein A, into sites within the Ad fiber. This motif was well tolerated when inserted into the HI loop of Ad5, resulting in negligible disruption to fiber trimerization. Using this approach, high affinity conjugation of human monoclonal antibodies to the tumor marker epidermal growth factor receptor (EGFR) [274,277], CD40 and CD40L [267], have been shown to result in infectivity enhancement in cells which express the cognate target receptor. Furthermore, this strategy has been expanded to allow targeting to neuronal cell adhesion molecule and the α7 integrin subunit, which are expressed on differentiated primary human myoblasts [274]. Retargeting of knobless Ad5 vectors has also been achieved by incorporating such Ig-binding motifs in a vector background featuring a truncated shaft and heterologous NRP trimerization domain [277]. Henning and colleagues tested the suitability of staphylococcal protein A IgG-binding motif, in addition to an alternative epitope, the C2 domain from streptococcal protein G (as it binds to a broader range of IgG subclasses) for conjugation of a range of antibodies directed to specific targets. These included monoclonal antibodies directed against major tumor antigens, including EGFR, Her2/neu and prostate-specific membrane antigen (PSMA), which successfully mediated CAR-independent cell transduction [277].

Earlier in this review, we described studies by Campos and colleagues who genetically incorporated the BAP peptide, derived from a bacterial transcarboxylase enzyme, into HVR5 of the Ad5 hexon, fiber, or pIX, to create metabolically biotinylated vectors [222]. This strategy was designed to facilitate the capsid site-selective conjugation of biotinylated retargeting ligands using a tetrameric avidin bridging system [132]. Retargeting has been achieved through the conjugation of antibodies directed against CD59, CD71, transferrin, EGFR [278] or cholera toxin B to fiber-modified BAP vectors, resulting in enhanced infection in CAR-negative carcinoma cell lines [222]. However, similar attempts using the pIX or hexon modified BAP-derivatives were shown to be ineffective, which the authors proposed to be due to defective endosomal escape or nuclear trafficking of the modified vectors [222].

Bispecific “adenobodies” are diabodies which possess dual selectivity, firstly for the target receptor, and secondly for the virus itself [279]. This can be exploited so that the bivalent moieties simultaneously bind native Ad receptor tropism determinants (e.g. the knob domain), thus facilitating the ablation of native tropism, while redirecting the transductional capacity of the vector. Bifunctional antibodies conjugated to Ad5 include those simultaneously directed towards PSMA [280], CEA [281], high molecular weight melanoma-associated antigen HMWMAA [270], EGFR [282], the endothelial cell surface protein endoglin [268] or Ly-6D [283]. Using this type of approach, enhanced *in vivo* targeting to pulmonary vasculature was achieved via conjugation of a bispecific diabody, 9B9 which is simultaneously targeted to the virus knob domain and the angiotensin-converting enzyme (ACE), which is preferentially expressed on pulmonary capillary endothelium [284–286]. Importantly, using a therapeutic derivative of the ACE-retargeted vector which encoded endothelial nitric oxide synthase (eNOS), the authors demonstrated that selective overexpression of eNOS in the lung endothelium resulted in a sustained hypotensive effect in a stroke-prone spontaneously hypertensive rat (SHRSP) animal model [284].

With a similar strategy in mind, Ab-sCAR ectodomain fusion proteins have been assessed as adapters and have demonstrated enhanced *in vitro* retargeting to c-erbB-2 [287], EGF [288] and CD40 [289] in a CAR-independent manner. Successful *in vivo* targeting to CEA-expressing tumors has been achieved following intravenous delivery of Ad5 conjugated to a bifunctional sCAR-anti CEA, scFv fusion complex [272]. Encouragingly, this enhanced tumor retargeting was accompanied by dramatic reductions in liver transduction. Additionally, Harvey and colleagues demonstrated that sCAR-fusion proteins retargeted to urokinase-type plasminogen activator receptor (uPAR) and EGFR, improved the transduction of a number of carcinoma cell lines [271]. Furthermore, in the same study the EGFR-targeted vector significantly delayed tumor growth in a murine xenograft model [271]. More recently, a novel strategy to exploit the high affinity interaction between FX and the Ad5 hexon for adapter-based retargeting has been described [273]. The conserved γ-carboxyglutamic acid (Gla) domain within coagulation factor X is responsible for binding to the hypervariable regions of the Ad5 hexon. Chen and colleagues generated FX-derived, Gla domain scFv-fusion proteins directed against the tumor targets HER2 and EGFR, or towards the stem cell marker, ATP-binding cassette protein G2 (ABCG2). These FX-scFv fusion proteins, complexed with Ad5, resulted in increased infection and cytotoxicity of tumor cells *in vitro* and *in vivo*. However, somewhat unexpectedly, these Gla-fusion proteins did not result in reduced liver transduction following intravenous delivery. This possibly raises questions about the *in vivo* stability of the complex, or whether endogenous FX levels still affected the virus tropism [273]. Indeed, this hypothesis was proposed by the authors and they further supported this by demonstrating that pre-treatment of animals with warfarin (to deplete coagulation factors) resulted in a significant reduction in liver transduction.

Applications for adapter-ligand based complexes currently are limited, since they do not meet human gene therapy requirements, a result of their low yield, often heterogeneous viral populations and possible lack of stability *in vivo*. They do, however, provide valuable evidence that such retargeting strategies can enhance gene delivery in a CAR-independent fashion, and perhaps further pharmacoanalysis and confirmation of complex stability may enhance their future clinical utility.

### 2.4. Summary of Retargeting Efforts

Adenoviral vectors are currently the most widely used viral vectors for gene therapy, with cancer (64.5%), cardiovascular disease (8.7%) and monogenic disorders (7.9%) being the most common disease targets (http://www.wiley.co.uk/genmed/clinical). In recent years, extensive pre-clinical validation of retargeted adenoviral vectors has highlighted their superior efficacy over unmodified Ad5-based vectors [290–292], prompting their use in clinical trials and in compassionate use schemes for the treatment of cancer [293–295]. Modified Ad vectors currently undergoing clinical assessment in humans include those containing an RGD motif within the HI loop of the Ad5 fiber, and Ad5 vectors which have been pseudotyped with the knob domain from Ad3 (see Table 2). To date, these vectors have been well tolerated in patients, with observed side effects being mild to moderate fever, transaminitis, thrombocytopenia and hyponatremia. More importantly, these retargeted vectors have displayed promising anti-tumoral activity [294,295]. Further assessments of tropism-modified vectors will be required to better understand dose-limiting, off-target interactions, which may be of critical importance in patients. Consequently, such studies will help to improve the safety and efficacy of retargeted Ad vectors in their development as clinical therapeutics.

## 3. Transductional Detargeting Strategies

Intravascular delivery of Ad5 leads to a complex series of interactions between viral capsid proteins and a range of host components. These include interactions with coagulation factors [63,65,66], resident macrophages [83,296,297], complement [92,298], blood cells [81,82] and neutralizing antibodies [206,299]. Ad5 displays rapid blood clearance kinetics following *iv* delivery in mice, with a half-life of less than 2 minutes [74], due to the non-specific sequestration of Ad5 in Kupffer cells [73]. This scavenging by hepatic macrophages leads to a nonlinear dose response for hepatocyte transduction [296]. The rapid clearance rate of Ad5 is a limiting factor for retargeting strategies, which aim to increase blood persistence in an attempt to improve bioavailability for *in vivo* targets. Therefore, optimally designed platform vectors for retargeting should feature modifications to avoid not only native tropism, but also the reticuloendothelial system, circulating antibodies (IgM and neutralizing), blood cells and coagulation factors.

### 3.1. Transductional Detargeting by Ablation of Native Tropism

Ablation of CAR-binding determinants was once considered an essential strategy for refining the broad tropism of Ad5 *in vivo*. This was based on the assumption that the two-step *in vitro* entry pathway for Ad5, via CAR and ανβ3/ανβ5 integrins, was also relevant *in vivo* following *iv* delivery. However, various studies have demonstrated that ablating native Ad5 tropism interactions (CAR and/or αν-integrins) has little effect on the tropism of intravascularly delivered Ad5 *in vivo* [75,300–305]. For example, Martin and colleagues demonstrated that simultaneous ablation of CAR and integrin-binding determinants did not reduce genome accumulation or transgene expression in the liver [303].

This was later explained by the finding that coagulation factor X was the principal determinant of hepatocyte transduction [61,66]. Nonetheless, the development of CAR-independent targeting strategies is still an important consideration in the design of Ad5-based vectors for applications in human disease. CAR-binding reportedly activates the inflammatory response to Ad5 in epithelial cells [306], and in hepatic tissue following retro-orbital administration [307]. Although the tissue distribution of CAR in humans has not been well characterized, it has been shown to be expressed in cardiac/skeletal muscle [308], as well as on human erythrocytes [81,82]. Furthermore, expression of CAR in the heart is also believed to facilitate viral myocarditis [309], an inflammatory cardiomyopathy often caused by viruses which have a tropism for CAR (e.g. adenoviruses and Coxsackievirus B viruses) [310]. Recently, a specific protein isoform of CAR has been localized to the apical surface of human airway epithelia [311]. CAR mRNA also has been detected in the heart, testis, small intestine, pancreas, prostate, liver, kidney and brain [32,312]. Importantly, the discovery that human, but not murine erythrocytes can aggregate Ad5 through CAR-binding is particularly relevant, as this can impede targeted delivery by sequestering virus in the circulation, as well as contributing to toxicity [75,81,82]. This has particular relevance when choosing suitable animal models in which to study the effects of *iv* delivered therapeutic Ads. For these reasons, it is important to consider that the localization of CAR in humans may impact the selectivity/toxicity of targeted delivery and serves to further highlight the necessity for developing CAR-independent retargeting strategies.

The precise molecular determinants for CAR binding have been described previously [28,30,31]. The fiber of Ad5 exists as a homotrimer, and the topological arrangement of the knob monomer is as an eight-stranded antiparallel β sandwich, with interspersing loop regions [313]. The loop regions vary from 8–55 aa residues and are designated as the AB, CD, DE, DG, GH, HI and IJ loop domains. Residues, Ser408 and Pro409 in the AB loop, Tyr477 in the DG loop and Leu485 in β-strand F, have been identified as the critical epitopes involved in a high affinity interaction with CAR [28]. Substitution mutations at these sites, S408E, P409A, Y477A and L485K, have been shown to effectively abolish the interaction with CAR [28,302]. Furthermore, CAR-binding mutations, S408E and P409A, have also been shown to prevent the agglutination of human and rat erythrocytes [75].

Proceeding from the original hypothesis, that ablation of the native receptor binding determinants of Ad5 would refine its broad tissue biodistribution, several studies reported the generation of vectors featuring mutations in the penton base RGD motif, or penton base mutants which were combined with CAR-binding ablation. These studies generated variable results, with some reports of successful reductions in liver tropism [75,148], whereas others concluded that penton modifications had no effect on hepatic transduction *in vivo* [304,314]. However, it is possible that these differences could also be attributed to species variations between mice, rats and non-human primates, the selected animal models in which these studies were performed.

The KKTK motif within the shaft of the Ad5 fiber has been proposed to promote direct binding to HSPGs [49,50]. Hepatocytes express high levels of HSPGs [315–317], thus it was thought that HSPG-mediated entry could contribute to the dramatic liver transduction observed following *iv* delivery of Ad5. Subsequently, various studies described significant hepatocyte detargeting in mice [318,319], rats [75,320] and non-human primates [314] as a result of exchanging the fiber shaft amino acids, KKTK for glycine-alanine-threonine-lysine (GATK). In contrast, when shaft-chimeric Ad5 viruses featuring long Ad31 or Ad41 shaft domains (lacking the KKTK motif) were generated, the liver accumulation, transduction and levels of pro-inflammatory cytokines produced were identical to Ad5 [321]. These data suggest that the KKTK motif itself is not responsible for a direct, receptor-mediated interaction with HSPGs. It now is believed that the shaft mutation confers rigidity/instability to the fiber, impairing the flexibility required for efficient receptor interactions [322]. Thus, the mechanism underlying the reduced liver tropism of these vectors is now thought to be due to the inefficient endocytosis, viral trafficking or endosomal escape [77,320]. Furthermore, it appeared for some time that transduction with KKTK mutants could not be rescued by ligand-directed retargeting; the incorporation of RGD-4C, or the endothelial targeting peptide QPEHSST, into the HI loop of the KKTK mutant vectors failed to produce efficient retargeting [318,320]. However, as we mentioned previously, direct modification of the KKTK motif in the fiber shaft to the integrin targeting motif RGDK resulted in efficient retargeting *in vitro* and *in vivo* [199]. More recently, successful retargeting has been achieved using the KKTK shaft mutant as a platform vector for the insertion of a peptide in the HI loop [323]. In this study, a helper-dependent adenovirus (HDAd) featuring the KKTK-GAGA modification, was detargeted from CAR and simultaneously retargeted via the insertion of a homing peptide for dorsal root ganglion (DRG) neurons [323]. Therefore, it appears that the success of this strategy is dependent on the biological capacity of the inserted ligand, and those peptides which are capable of promoting their own internalization would be most suitable candidates.

### 3.2. Transductional Detargeting by Ablation of “Bridging” Interactions

A prominent role for receptor-independent “bridging” interactions in directing the *in vivo* tropism of Ad5 has been discovered in recent years. Several studies have now demonstrated an important role of coagulation factors in directing liver gene transfer [63,65,66]. The first study, published by Shayakhmetov and colleagues, suggested that binding of coagulation factor IX (FIX) and complement (C4)-binding protein to the fiber knob domain could potentially “bridge” the viral capsid to cellular HSPGs and low density lipoprotein receptor-related protein (LRP) receptors on the surface of hepatocytes [65]. The authors described an Ad5 mutant (Ad*mut*), featuring a combination of mutations within the fiber knob domain which abrogated binding to FIX/C4BP *in vitro*, resulting in a reduction in hepatocyte transduction, hepatotoxicity and a failure to co-localize with Kupffer cells following intravenous delivery [65]. Subsequently, Parker and colleagues showed that the *in vitro* transduction of Ad5 could be enhanced by multiple homologous vitamin K-dependent coagulation factors including FVII, FIX, FX or protein C, but not by the divergent prothrombin FII or FXI [63]. Furthermore, when vitamin-K dependent coagulation factors were depleted *in vivo* using warfarin, a widely used anticoagulant drug which prevents the maturation and secretion of vitamin-K dependent zymogens by blocking γ-carboxylation, the hepatocyte transduction of a CAR-binding ablated Ad5 vector (AdKO1) was reduced ∼300 fold compared to untreated animals. Importantly, hepatocyte transduction could be rescued completely following *in vivo* complementation with physiological levels of FX; suggesting that an Ad5:FX interaction represented a novel mode of Ad5 uptake *in vivo*, which was independent of the primary Ad5 receptor determinant, CAR.

In an attempt to fully dissect out the precise mechanisms underlying this alternative “bridging” route of hepatocyte transduction, pilot studies investigated the interactions of unmodified, CAR-binding Ad5 [324], or Ad5 vectors pseudotyped with fibers from species D adenoviruses (Ad47, Ad33, Ad24, Ad45, Ad17 and Ad30) with FX [62]. *In vitro*, these Ad5 fiber-pseudotyped vectors bound to FX efficiently, as determined by surface plasmon resonance (SPR). Additionally, co-incubation of these vectors with FX resulted in enhanced FX-mediated cell binding and transduction of HepG2 cells. Therefore, these *in vitro* data suggested that the FX-Ad5 interaction was independent of fiber interactions, as fiber pseudotyping had no effect on FX-mediated infectivity. Subsequently, following three-dimensional (3D) cryo-electron microscopy reconstruction of the Ad5-FX interaction [66], the Ad5 hexon was identified as the key FX-binding capsid protein, with each trimeric hexon shown to form a complex with FX with a stoichiometry of one FX molecule per hexon. Furthermore, the conserved γ-carboxyglutamic acid (Gla) domain within FX was identified as the precise domain responsible for binding to the hypervariable regions (HVR) of the Ad5 hexon [61,66]. These findings prompted a systematic analysis of the FX-binding capacity of various human Ad species [61,66,325]. SPR analysis revealed distinct differences in FX-binding affinities of different Ad serotypes, with Ad5, Ad2 and Ad16 displaying high affinity binding to FX, whilst species D adenoviruses (including Ad48 and Ad26), failed to bind FX *in vitro* [61,66]. Using hexon-chimeric Ad5-based vectors in which some or all HVR loops of the hexon were substituted for the corresponding regions from Ad48 or Ad26, it was shown that hepatocyte transduction could be dramatically reduced following *iv* delivery [60,66]. Two independent studies corroborated these data, confirming that the Ad5 hexon-FX interaction was the critical determinant of hepatocyte transduction *in vivo* [61,64]. Using a HVR5-modified Ad5 vector featuring the insertion of BAP [222], Kalyuzhniy and colleagues demonstrated that hexon modifications could also abrogate hepatocyte transduction following intravenous delivery [61]. Similarly, following the generation of several hexon-modified vectors containing different peptides inserted into HVR5, Vigant and collaborators again showed that liver gene transfer was significantly reduced [64].

**Figure 5:**
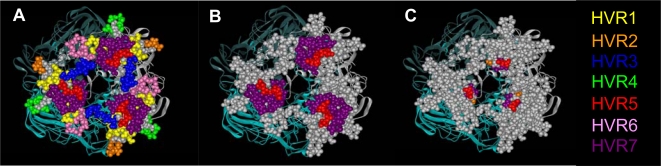
Top View of Adenovirus type-5 Hexon Protein. **(A)** All the hexon hypervariable regions (HVR) are highlighted in different colors, which are indicated on the right. **(B)** HVR5 (red) and HVR7 (purple) were identified as key domains involved in FX-binding. **(C)** Identification of critical FX-interacting amino acid residues within HVR5 and HVR7. Residues are as follows; highlighted in red, HVR5 epitopes T270P and E271G and highlighted in purple HVR7 epitopes I421G, T423N,E424S, L426Y and E451Q (in orange). This figure has been reproduced with permission. This research was originally published in Blood. Alba *et al.*, Identification of coagulation factor (F)X binding sites on the adenovirus serotype 5 hexon: effect of mutagenesis on FX interactions and gene transfer. Jul 2009; 114: 965 – 971. © the American Society of Hematology [60].

More recently, the critical domains and precise epitopes responsible for mediating the hexon-FX interaction have been mapped to hexon HVR5 and HVR7 [60]. In this study, cryo-electron microscopy was integrated with structural modeling (based on existing crystallographic data), to predict the putative interacting residues in the Ad5:FX complex. Initially, hexon chimeric vectors were generated which featured HVR5, HVR7 or HVR5+HVR7 substitutions with the corresponding HVR regions from Ad26 (which did not bind FX by SPR). These modified vectors failed to transduce hepatocytes *in vivo*. Subsequently, following the identification of these key residues, the authors used site-directed mutagenesis to introduce point mutations specifically at these sites (Figure 5). These modifications included amino acid substitutions in HVR5 (T270P and E271G) and HVR7 (I421G, T423N, E424S, L426Y and E451Q). Using SPR analysis, cell binding assays, *in vitro* transduction assays and *in vivo* studies to assess liver gene transfer, the authors confirmed the importance of these selected residues in mediating the high-affinity interaction with FX. Importantly, the point mutations identified within HVR7 were shown to play the most significant role in FX-binding and liver gene transfer than mutations introduced in HVR5. A single amino acid residue, E451, was found to be conserved among all FX-binding human Ad serotypes, while the residue Q451, was identified in non FX-binding Ad serotypes. Accordingly, it was shown that a single point mutation at this site, E451Q, was sufficient to ablate FX-mediated infectivity enhancement *in vitro* and *in vivo* [60].

With the aim of increasing the blood persistence and bioavailability of the virus for its target tissue, the experimental use of anti-coagulants, such as warfarin, has been employed in many tumor targeting studies. The level of success of such strategies has not been optimal, with no improvements, or even reductions in tumor uptake reported [149,326–328]. Furthermore, no increase in the tumor uptake of retargeted vectors, Ad5/3, Ad5-pK7 [328], Ad5 retargeted to ανβ6 [149] or Ad5-RGD-4C were observed following pre-treatment with warfarin [328]. However, the combination of coagulation factor and macrophage depletion expanded the therapeutic window of Ad-delivery [327,329]. Together, these data suggested that coagulation factors may play a role in tumor uptake *in vivo*, and that avoidance strategies could potentially impair efficient tumor transduction. However, a subsequent study demonstrated that the *in vivo* retargeting of fiber pseudotyped Ad5/35 to CD46+ liver metastases, was improved significantly when using X-bp to selectively inhibit FX [237]. Furthermore, the use of an alternative Ad serotype, Ad35, for which the hexon:FX binding affinity is ∼10-fold lower than it is for Ad5, increased gene transfer to the lung following *iv* administration in CD46-transgenic mice [325]. Based on these findings, it seems that hexon-modified vectors which are genetically ablated specifically for FX-binding could represent excellent platform vectors for retargeting strategies where avoidance of the liver is a prerequisite. The use of genetically modified vectors which avoid coagulation factors is likely to be more clinically applicable than attempting to administer immunocompromised patients with combinatory anti-coagulant/therapeutic Ad treatment regimes.

To date however, reports of hexon-modified, retargeted vectors which display targeting superior to Ad5 *in vivo* are limited. Hexon-modification strategies which have been assessed for tumor uptake include an oncolytic derivative of the Ad5BAP-modified vector (Ad-GL-HB), which was shown to have significantly reduced levels of hepatic transduction, decreased liver cell damage and increased dose-tolerance *in vivo* when compared to parental Ad5 [329]. This vector, Ad-GL-HB, maintained equivalent tumor transduction levels to Ad5, although it exhibited drastically improved tumor:liver ratios as a result of its limited hepatotropism [329]. Additionally, Vigant and colleagues reported similar results, with equivalent tumor transduction when comparing HVR5-retargeted Ad5 with unmodified Ad5 [64]. Alternatively, HVR-substituted vectors represent another attractive platform for the design of Ad-retargeting strategies, as they can simultaneously avoid coagulation factor binding, in addition to potentially permitting escape from anti-hexon NAb *in vivo* [206,330,331]. However, despite the successful generation and amplification of many hexon-chimeric Ad vectors [330–334], the exchange of hexon regions for those derived from alternative serotypes can be limited by the formation of non-viable virions [335]. A list of reported hexon modifications is shown in Table 3. For this reason, it may be preferable to generate retargeted vectors with defined point-mutations in the hexon for avoidance of coagulation factor binding. Using this approach, combining Ad35++ fiber pseudotyping [240] with FX-binding ablating mutations in the hexon, dramatically improved lung:liver ratios in macrophage-depleted CD46-transgenic animals [242].

### 3.3. Detargeting from the Reticuloendothelial System

The mechanisms which govern uptake of Ad vectors by resident hepatic and/or splenic macrophages are not clearly defined, and are currently believed to be due to scavenging activity. There is evidence to suggest that the knob domain of Ad5 may contribute to some extent to sequestration of Ad5 in macrophages. The Ad*mut* vector described by Shayakhmetov and colleagues, (featuring a CAR-binding ablation mutation and a TAYT deletion in the fiber knob), was reported to have reduced co-localization with Kupffer cells following intravenous delivery [65]. More recently, it was proposed that SR-A was responsible for the accumulation of Ad5 in macrophages. *In vitro*, this was demonstrated using the murine macrophage-like cell line, J774 and primary rat Kupffer cells [89]. Interestingly, pre-incubation with recombinant Ad5 knob protein was capable of inhibiting the entry of Ad5 in primary rat Kupffer cells. Subsequently, the authors also confirmed that SR-A contributed to the uptake of Ad5 *in vivo* following *iv* delivery, and that the knob domain was potentially involved in mediating this interaction. Pre-injection of mice with SR-A ligand, poly(I), partially precluded Kupffer cell scavenging in the liver. Furthermore, pre-incubation of Ad5 with an anti-knob antibody dramatically reduced the amount of virus detected in co-localization with hepatic macrophages, as determined by immunohistochemistry.

Alternatively, it is likely that diverse Ad-species have different overall electrostatic properties. This may impact on their uptake by scavenging receptors on KCs, which preferentially recognize negatively charged materials [74,336,337]. Therefore, the manipulation of Ad5 vectors by pseudotyping suitable fibers and/or capsid proteins may help to generate chimeric vectors which could potentially alter the *in vivo* characteristics of the predominantly negative Ad5 particle [74]. More recently, opsonization by complement (C3 and C4), in combination with natural IgM antibodies, has been proposed as an alternative mechanism for the uptake of Ads by scavenging receptors on Kupffer cells *in vivo* [83]. Interestingly, the electrostatic characteristics of Ad5 can also dictate the extent of recognition by serum proteins, including complement [336,338]. The identification of factors which determine uptake by macrophages could potentially be exploited in the future, allowing avoidance of scavenging and subsequent degradation of therapeutic vectors. Such strategies, if coupled with current advancements in hepatocyte detargeting would result in gene delivery vectors with increased clinical utility.

### 3.4. Transductional Detargeting and Retargeting by Chemical Modification

An alternative method for detargeting Ad vectors, which bypasses the requirement to introduce multiple genetic modifications into capsid proteins, involves utilizing polymers to chemically modify the capsid. The mainstay polymer utilized for this purpose is based on polyethylene glycol (PEG), a monovalent hydrophilic polymer which covalently attaches to the virus capsid most commonly via free surface reactive amine groups [339–341] or via introduced disulfide groups [342,343]. The basic form of PEG is an uncharged linear polymer composed of repeated subunits of (CH_2_CH_2_O), typically with a molecular weight ranging from 200 to 40,000, and containing either a single (semitelechelic), or two (bifunctional) terminal reactive groups.

Various strategies can be used to generate PEG-modified constructs. These include the use of activated monomethoxypolyethylene glycol (MPEG), which is coupled to proteins using a triazine ring [344], tresylmonomethoxypolyethylene glycol (TM-PEG) which preferentially reacts with *ɛ*-amino terminal of lysines [341], or succinimidyl succinate PEG (SS-PEG) which uses the amino reactive *N*-hydroxysuccinimide (NHS) ester of PEG succinate to couple to target proteins [345]. PEGylation of protein compounds has long been established as an effective means of increasing the solubility and circulatory half-life of proteins in the bloodstream by preventing proteolytic degradation, whilst simultaneously reducing antigenicity and immunogenicity. A selection of these attributes can also be extended to PEGylated Ad5. This is because such monovalent polymers form a “polymeric cloud” around the vector, thus providing extensive masking of the capsid, potentially shielding the vector from undesirable *in vivo* interactions with native receptors, coagulation factors or NAbs. However, PEGylation of biologically active molecules is often limited by reductions in their activity. Therefore, whilst PEG confers improvements on Ad pharmacokinetics *in vivo*, the use of multivalent hydrophilic polymers (*i.e.* bearing multiple reactive groups) can confer Ad vectors with additional benefits in terms of stability.

Vectors modified with polymers such as those based on poly[N-(2-hydroxypropyl)methacrylamide] (pHPMA) show substantially increased biological stability, with marked improvements in systemic circulation times [346]. Modification of vectors using multivalent (e.g. HPMA), instead of monovalent (e.g. PEG) polymers, offers a degree of lateral stabilization to the vector, providing more closely associated shielding, a “polymeric cage” rather than a “polymeric cloud”. Once vectors are modulated in this manner, the two-step Ad5 transduction pathway (via CAR/integrins) can also be abolished, subsequently setting the challenge of efficiently retargeting the tropism of the complex via the introduction of suitable targeting ligands. Using suitable chemistry, further modification strategies can be devised to permit dissociation of the polymeric coat from the virus complex following successful uptake into target cells, facilitating effective trafficking to the nucleus for subsequent transgene expression and/or viral replication.

#### 3.4.1. Tropism Detargeting Adenovirus by Chemical Modification

Initial studies, performed in 1997, established a means of complexing Ad5 with cationic polymer or lipid molecules [347]. Subsequently, Chillón and colleagues pioneered strategies using the cationic lipid, GF-67, to noncovalently couple PEG to the Ad5 capsid [339]. The authors demonstrated that GF-67-PEGylated Ad5 successfully evaded antibody binding *in vitro*; however the strategy failed to provide significant protection from NAbs when delivered *in vivo* in pre-immunized animals [339]. O’ Riordan and colleagues quantified the association of PEG to the Ad capsid, estimating that approximately 18,000 PEG molecules covalently attached to the Ad5 capsid via the major capsid proteins hexon, penton base and fiber, whilst the core proteins remained unmodified [341]. Furthermore, the authors demonstrated that this approach enabled the evasion of NAb *in vitro*, and more importantly, this was the first example that the vectors retained their capacity to transduce *in vivo* following intratracheal delivery of PEGylated Ad5 in mice pre-immunized with Ad5. Subsequently, Croyle and colleagues extended these studies, comparing PEG molecules with a range of activation linkers and determining optimal PEGylation approaches to maintain adequate virus infectivity [348].

The vast majority of early studies on chemical shielding of Ad vectors focused on the evasion of NAb and innate immune responses (these approaches will be discussed in *Section 3.4.3.*). However, more recently, a focus to adenoviral gene therapists has been to exploit these vectors for avoidance of hepatocyte transduction. Accordingly, a number of studies have shown that the size of PEG molecule coupled to Ad5 can impact on its biodistribution profile *in vivo* following *iv* delivery. It has been shown previously that PEGylation of Ad5 with small PEG molecules (e.g. 2–5 kDa) has no discernible effect on hepatocyte transduction, despite the capacity to efficiently detarget *in vitro* [349,350]. However, this is not surprising as it is now clear that detargeting from native *in vitro* receptors is redundant in terms of affecting *in vivo* liver transduction, which is mediated primarily by FX. In contrast to small PEG modifications, PEGylation using larger PEG molecules (20–35 kDa) can significantly reduce liver transduction [350,351]. Interestingly, Hofherr and colleagues compared the interaction of coagulation factors VII, FIX and FX with PEGylated Ad5 vectors, modified by conjugation of 5 kDa or 35 kDa PEG molecules [351]. The authors demonstrated that although these vectors both retained the ability to interact with coagulation factors *in vitro*, in particular with FIX and FX, only Ad5 modified with 35 kDa PEG had significantly reduced liver transduction following *iv* delivery. However, liver transduction with both vectors (albeit already significantly lower with the 35 kDa-modified Ad5) was shown to be reduced following depletion of coagulation factors using the anti-coagulant warfarin, demonstrating that these vectors maintained an interaction with FX *in vivo*. Therefore, it was hypothesized that the size of the 35 kDa-modified Ad5 was potentially contributing to its reduced hepatic transduction, possibly due to liver fenestrae size limitations or increased stability of the virus complex *in vivo* [351].

In addition to these PEG modification strategies, polymer coating of Ad5 using HPMA has also been shown to result in reduced hepatic transgene expression [346]. Green and colleagues demonstrated that coating Ad5 with HPMA led to increase blood persistence and resulted in a dosedependent reduction in liver uptake, with at least 100-fold reduced liver transduction following *iv* injection of virus at the highest dose of virus (6 × 10^11^ vp) [346]. This was accompanied by reduced toxicity, as determined by minimal transaminase elevations, which were comparable to the level detected in control, untreated animals. The multivalent nature of HPMA permits multi-site attachment to the virus surface, resulting in partial (∼70%) shielding of the capsid under standard conditions [352]. This not only negates cross-linking of vector particles following modification, but more importantly, due to the multivalent nature of this polymer, excess unreacted NHS-ester groups remain available for subsequent incorporation of amine-containing targeting ligands.

#### 3.4.2. Tropism Retargeting Adenovirus by Chemical Modification

The extended plasma kinetics observed with chemically modified Ad vectors makes them particularly attractive for tumor targeting applications. This is due to the potential for increased bioavailability [346], or passive uptake of such vectors by tumor tissue [353,354] by means of enhanced permeability and retention (EPR) as a result of leaky tumor vasculature [355,356]. However, shielding of Ad capsid proteins following chemical modification frequently results in vectors which have reduced transduction efficiency, often as a result of the occlusion of native receptor binding epitopes (e.g. CAR and/or integrin binding motifs). Therefore, an advantageous property of vectors modified by chemical coupling of PEG or PHMA, is the possibility to incorporate targeting ligands into the polymeric “cloud” which can confer an alternative tropism to the vectors.

Initially, Romanczuk and colleagues demonstrated the feasibility of such an approach using a bifunctional PEG molecule which featured both amine (reactive with lysine residues on the virion surface) and sulfhydryl reactive groups (selectively reactive with cysteine residues incorporated within a targeted peptide sequence) [357]. The authors described a peptide (sss.17) identified by phage biopanning, which displayed increased binding to primary normal human bronchial cells [357]. The corresponding PEGylated, retargeted Ad5 vector mediated enhanced, ligand-dependent transduction (which was independent of the fiber knob domain) in well-differentiated human airway epithelial cells which exhibited a ciliated morphology. Furthermore, the chemically retargeted vector was shown to be less susceptible to NAbs *in vitro* [357]. Again, through the use of heterobifunctional forms of PEG (that is PEG containing two different reactive groups), Lanciotti and colleagues were able to incorporate a genetically engineered form of basic fibroblast growth factor (bFGF) via a thiol reactive maleimide group [358]. The resulting bFGF retargeted vectors demonstrated CAR-independent, enhanced gene transfer *in vitro* and *in vivo* using a tumor xenograft model when compared to nonretargeted PEGylated Ad5. Furthermore, both the bFGF retargeted and PEGylated adenoviral vectors transduced the liver and spleen with approximately one log lower efficiency compared to unmodified Ad5 [358]. Thus, the use of heterobifunctional PEG enabling the presentation of high affinity ligands to retarget shielded PEGylated Ad vectors, represent a promising approach to generating efficiently retargeted vectors for *iv* delivery.

Retargeting efforts using chemically modified Ad vectors are not limited to PEGylation strategies. Early studies demonstrated that *in vitro* retargeting via basic fibroblast growth factor (bFGF) or vascular endothelial growth factor (VEGF) could be achieved in a ligand-specific manner following coupling to multivalent HPMA-modified Ad5 [352]. The authors initially chose these ligands based on reported success following retargeting Ad5 via FGF [359], or due to the proposed compatibility between the Ad entry pathway and the ligand-binding induced endosomal pathway of VEGF [360]. Parker and colleagues also evaluated a HPMA-modified Ad5 vector which was retargeted via the incorporation of the SIGYPLP oligopeptide [361]. This retargeted vector restored efficient transduction *in vitro* (when compared with HPMA-modified, but non-retargeted Ad5), and enhanced the level of transgene expression in human umbilical vein endothelial cells (HUVECs) in accordance with its previously described selectivity for endothelial cells [156].

In terms of tumor targeting efforts using HPMA-modified vectors, Stevenson and colleagues described the successful covalent linkage of a laminin-derived peptide (SIKVAV) to a HPMA-modified Ad5 vector [362]. The expression of α6-integrin heterodimers is reported to be altered in human carcinoma cells, and increased expression of α6β1 (a natural receptor for laminin) [364], has been reported to contribute to a migratory and invasive phenotype [364,365]. The addition of the SIKVAV targeting ligand to polymer coated Ad, restored its transductional capacity in a CAR-independent and ligand concentration dependant manner [362]. Moreover, *in vivo* delivery of this vector maintained efficient tumor transduction levels when compared to unmodified, non-polymer coated Ad5. The hepatic transduction of HPMA-SIKVAV-Ad5 was also significantly reduced compared to unmodified Ad5, thus improving tumor:liver ratios. A subsequent study described the successful modification of polymer coated, wild-type Ad5, through the incorporation of murine epidermal growth factor (mEGF), which selectively targets the EGF receptor [366]. Importantly, this vector resulted in an improved therapeutic outcome using an intraperitoneal (*ip*) SKOV-3 ovarian xenograft model. The authors showed that *ip* delivery of mEGF-HPMA-Ad5 improved median survival when compared with non-retargeted HPMA-modified Ad5 [366]. More recently, the authors significantly improved on these studies by retargeting HPMA-Ad5 to EGFR by coupling an anti-EGFR antibody, cetuximab, a more clinically feasible targeting ligand than bEGF or mEGF (as these are potential mitogens) [367]. Again, this chemically modified vector resulted in significantly improved survival in mice bearing *ip* SKOV3 xenografts, to a level comparable to wild-type Ad5. Despite equivalent levels of anti-tumoral efficacy when compared with wild-type Ad5, retargeted vectors which are simultaneously modified using reactive polymers, are still considered advantageous as they exhibit reduced hepatic transduction and inflammatory toxicities *in vivo*, qualities which Ad5 vectors lacking chemical shielding do not possess [367].

More recently, a number of novel studies have sought to overcome some of the current limitations associated with covalent attachment of targeting ligands to polmer-coated Ads. These limitations can include loss of the biological efficacy of the targeting ligand, conformational restrictions to optimal ligand presentation or diminished polymer-coating following the addition of targeting ligands [368]. Wilemsen and colleagues described a multivalent reactive HPMA-modified Ad5 vector which featured an α-bungarotoxin binding peptide (BTXbp), which has a nanomolar binding affinity for its cognate protein (BTX), thus permitting non-covalent addition of BTX fusion proteins. The authors used a recombinant anti-PMSA scFv antibody fragment, featuring the BTX binding domain, to demonstrate the feasibility of this approach. The retargeted, polymer-coated virus displayed selectivity for PSMA-expressing prostate carcinoma cell lines *in vitro*. However, this retargeting strategy was shown to slightly compromise successful transgene expression, despite equivalent levels of cell binding when compared to unmodified Ad5. Despite this, the HPMA-BTXbp capsid modification represents a flexible system for the conjugation of a wide array of BTX retargeted fusion proteins. Further assessment of this type of vector using alternative retargeting ligands (perhaps those ligands which are capable of promoting their own internalization) may yield interesting results in the future. In another study, Wang and colleagues have successfully modified Ad capsids for the first time using chitosan, a natural cationic polymer [369]. The approach involved using the reactive cross-linking reagent *N*-[γ-maleimidobutyryloxy]succinimide ester (GMBS) to generate maleimide-modified Ad5. This vector was subsequently conjugated with chitosan-SH, which conferred an almost neutral surface charge on the negatively charged Ad5 virion, without significantly changing its physical size [369]. Chitosan is thought to be an attractive molecule for mucosal drug delivery approaches due to its ability to adhere to mucus, and to traverse through mucosal barriers [370]. Experiments performed on pre-immunized rats indicated that Ad-GMBS-ChiSH displayed enhanced transgene expression throughout corneal epithelial cells, suggesting that the vector was increasingly resistant to NAb responses *in vivo*. Therefore, the authors proposed that chitosan-modified Ad5 would represent a useful platform vector for delivery to the ocular surface.

In summary, successful retargeting can be achieved with chemically modified Ads using a variety of ligands including growth factor molecules such as bFGF [352,371] and VEGF [352], scFv fragments directed against EGFR [366,367], sugars [372] and various peptide ligands capable of binding cellular receptors [361].

#### 3.4.3. Avoidance of Immune Responses Following Chemical Modification of Adenovirus

The use of chemical modification strategies to shield the Ad capsid confers significant improvements in adenoviral pharmacology, not only in terms of limiting the extent of hepatic damage, but also through the evasion of NAb and by reducing the induction of innate immune responses directed against the vector. Taken together, these characteristics may enable the generation of safer vectors for *iv* gene transfer.

As stated in the previous section, the main emphasis in early reports of chemical modification strategies for Ad5, focused on the evasion of pre-existing NAb, or indeed the avoidance of activating humoral immunity. However, in addition to the evasion of NAb, PEGylation of Ad vectors has the potential to limit innate anti-viral immune responses following *iv* administration. In a 2005 study, Mok *et al.,* demonstrated that PEGylated Ad vectors induced lower serum interleukin-6 (IL-6) levels 6 h post-injection than unmodified Ad following administration of 3 × 10^11^ vp [349]. However, liver damage gauged by serum transaminase levels remained unchanged. Interestingly, Mok and colleagues also comparatively assessed the uptake of fluorescently labeled Ad5, or labeled, PEGylated Ad5 vectors by KCs following *iv* delivery [349]. The authors found a decreased KC association of fluorescently labeled PEGylated vectors when compared with labeled Ad5. Furthermore, when murine macrophage cells (RAW267.4) were incubated with PEGylated vector particles *in vitro* production of IL-6 was decreased. These differences may be due to alterations to the overall electrostatic charge of Ad5 as a result of the chemical modification process. Alternatively, several groups have proposed that PEGylation modifies critical epitopes in the Ad5 fiber which are involved in recognition/uptake by macrophages [348,349]. This hypothesis is in accordance with more recent reports which have suggested a role for the fiber knob in uptake via SR-A [89].

In a separate study, Croyle and colleagues reported similar findings using a lower dose of PEGylated Ad (1 × 10^11^ vp) which was injected systemically [348]. The authors observed decreased levels of serum IL-6 and IL-12 6 h post-injection when compared with unmodified Ad5, whilst also noting significantly decreased levels of serum alanine aminotransferase (ALT) and aspartate aminotransferase (AST) liver transaminases, indicative of reduced hepatic damage following chemical modification. This latter finding was apparently in contrast with the former study by Mok and colleagues [349]. This discrepancy may be related to the lower dose of administered virus, the different mouse strains utilized or the specific formulations of PEG used in these respective studies. Another important observation noted by Croyle and colleagues was that mice administered with PEGylated Ad showed no decrease in platelet counts, whereas it is well established that Ad5 vectors induce transient thrombocytopenia. Thus, these studies suggested that PEGylation may also help prevent the onset of thrombotic conditions such as disseminated intravascular coagulation (DIC), following intravascular administration of Ad vectors. Further evidence for the preferential effects of PEGylation on Ad-induced innate immunity was provided by De Geest and colleagues [340]. In agreement with the aforementioned studies, they noted that PEGylation of Ad5 resulted in significantly lowered serum levels of IL-6 during the acute phase, whilst further noting that the mRNA levels of a variety of other cytokines were reduced in the liver 6 h post-injection [340]. The authors also evaluated the effects of PEGylation on vector biodistribution, noting that reduced levels of IL-6 correlated with significantly reduced accumulation of PEGylated Ad5 in the spleen, the major site of IL-6 production. This is in agreement with another study, using fiber-modified but not chemically modified Ad vectors, which reported that vectors which exhibit reduced splenic uptake display limited induction of IL-6 [373].

#### 3.4.4. Summary of Chemical Modification Strategies

Although chemical modification strategies hold promise for future retargeting strategies, they may have limitations for certain applications, namely the therapeutic treatment of cancer using oncolytic vectors. This is due to the fact that the retargeted polymer coat is not heritable, and therefore, unlike genetic modification strategies (which propagate the modification through each round of viral replication) progeny virions lack the potential for expansive oncolysis via the incorporated targeting ligand. This property would be undesirable for oncolytic vector design which hinges on maximizing intra-tumoral spread following virus replication. Furthermore, the reduced transduction efficiency (or simply equivalent transduction to unmodified Ad5) often observed with chemically modified vectors suggests that further optimization is required in order to generate vectors with dramatically improved uptake in target tissue *in vivo*. This will require the evaluation of candidate ligands which retain their biological efficacy following the chemical coupling process, which are efficiently presented and accessible for target receptor binding, allow subsequent virion internalization and which permit successful dissociation and/or delivery to cellular compartments which are conducive to transgene expression. However, continued advances in polymer chemistry to incorporate “stimuli-responsive” elements into the polymer to permit its removal following arrival at or within the target cell, for example incorporation of pH responsive elements which activate at decreasing pH (such as experienced within the endosome) or within reducing environments (such as the intracellular environment), are also being synthesized [374]. These approaches, coupled with the inclusion of suitable, high-efficiency peptide or antibody retargeting will improve the future development of chemically modified Ad-retargeting platforms with clinical potential.

### 3.5. Summary of Detargeting Strategies

It is now evident that the distinct hepatotropism observed with Ad5 in murine models is mediated through an interaction between the Ad5 hexon and FX [61,66]. Furthermore, evidence suggests that this “bridging” pathway may also be relevant in other animal models, including Syrian hamsters [375,376] and rats [255]. However, the role of this interaction in determining liver transduction in humans has not been characterized. The fenestrae of the sinusoidal endothelial cells in the livers of mice are relatively large (∼140 nm) and easily accommodate the smaller Ad5 particle (<100 nm). This permits rapid access to hepatocytes and to the space of Disse [377,378]. Conversely, the smaller endothelial fenestrae (∼107 nm) in humans may be more restrictive to Ad delivery [378,379], as fenestrae size is thought to be an important determinant of hepatocyte transduction [377]. Murine and human FX display high amino acid sequence homology [380], and both bind to Ad5 with high affinity [61,66,381]. Evidence supporting the relevance of the FX-hexon functional interaction has been demonstrated *in vivo* using human FX to rescue hepatocyte transduction in warfarinized mice [63]. Whether or not this interaction plays a dominant role in limiting the efficacy of clinically used Ads, or inducing toxicity in humans, remains to be determined. However, it is well established that the coagulation cascade is intrinsically linked with cancer and importantly, elevations in FX are frequently detected in patients with solid tumors [382–384]. Furthermore, acute transient transaminitis is a frequently reported contraindication in clinical trials studies using Ad5 [385–388]. Therefore, it is worth considering that the Ad5-FX interaction may well be especially relevant in immunocompromized, human patients undergoing oncolytic Ad5-therapy.

Recent evidence has also implicated native receptor binding determinants (CAR/integrins) in the potent activation of cytokines and chemokines by Ad5 [37,72,80,306,307,389]. These interactions can induce various signal transduction pathways including p38MAPK, p44/42MAPK (ERK1/2), PI3K and nuclear factor kappa-light-chain-enhancer of activated B cells (NF-κB). The finding that Ad5 can mediate binding to CAR on the surface of human erythrocytes [82], has been proposed to limit its targeting potential *in vivo* [81]. Additionally, Ad5 binding to CAR has been identified as a key event leading to the activation of pro-inflammatory cytokine transcription in respiratory epithelial cells *in vitro* [306], and has been associated with the induction of cytokine transcription *in vivo* [307]. Furthermore, an Ad5 interaction with the integrin subunit -β3, has also been shown to promote the activation of IL-1α in splenic marginal zone macrophages *in vivo* [389]. Collectively, these factors support the necessity for the incorporation of detargeting modifications, not limited to the ablation of coagulation factor binding, into tropism modified Ad-vectors. It is likely that the current detargeting criteria are not exhaustive, and further interactions will be uncovered in the future which will also require consideration and inclusion into strategies for Ad-based therapeutics.

## 4. Final Concluding Remarks

Several major challenges which limit the translational potential of adenoviral vectors, especially when attempting to achieve targeting following *iv* delivery, currently exist. A significant proportion of the data regarding the *in vivo* biodistribution, toxicity and efficacy of adenoviral vectors refer to studies performed in small animal models, namely mice. Undoubtedly, these studies have led to increased knowledge in the field and have influenced the future direction of Ad-based therapeutic strategies. However, the translational relevance of many of these findings requires further validation. Species variations in innate immune responses, permissiveness for viral replication, differences in hepatic micro-anatomy, differential interactions with blood cell populations, native receptor expression, in addition to the presence of pre-existing neutralizing immunity all contribute to the broad spectrum of Ad responses observed in pre-clinical animal models. However, emerging data from clinical trials are helping to direct future pre-clinical efforts, and indeed influence the choice of animal models in which to study adenoviral responses.

Despite the many limitations, the advances which have been made in recent years, particularly in terms of detargeting Ad5 from its inherent hepatotropism, have been significant. Combinatory retargeting approaches using genetic hexon-modified platform vectors are currently in their infancy, and it is clear that studies which aim to further characterize the *in vivo* biodistribution of these vectors will provide substantial foundations for the design of optimized retargeting strategies. Moreover, advances in the identification of novel disease-specific biomarkers, combined with technical developments and novel approaches to retargeting strategies, will permit the selection of customized vectors with improved efficacy. As a whole, achieving truly retargeted Ad-delivery, devoid of undesirable *in vivo* interactions is becoming a more realistic prospect for the near future.

## Figures and Tables

**Table 1:** Summary of Human Adenoviruses (*Mastadenovirus*).

**Classification Scheme**				**Receptor Usage**	**Tropism**
**Species**	Serotype/Type*	**Haemagglutination Groups**	**Percent G:C**		
A	12, 18, 31	IV (Little or no agglutination)	48–49	CAR*^a^*	Enteric, respiratory
B1	3, 7, 16, 21, 50	I (Complete agglutination of monkey erythrocytes)	51–52	CD46*^b^*, CD80*^c^*, CD86*^c^*	Renal, respiratory, ocular, urinary tract (UT)
B2	11, 14, 34, 35, 55^†^	I	48–49	CD46*^b^*, CD80*^c^*, CD86*^c^*, Receptor ‘X’*^d^*	Renal, respiratory, UT
C	1, 2, 5, 6	III (Partial agglutination of rat erythrocytes)	57–59	CAR*^a^*, HSPG*^e^*, αMβ2*^f^*,αLβ2*^f^*	Respiratory, ocular, lymphoid
D	8, 9, 10, 13, 15, 17, 19, 20, 22–30, 32, 33, 36–39, 42–49, 51, 53^†^, 54^†^	II (Complete agglutination of rat erythrocytes)	57–61	sCAR*^a^* (Ad9, Ad19p), CD46*^b^*, sialic acid*^g^* (Ad37, Ad19a, Ad8)	Enteric, ocular (keratoconjunctivitis)
E	4	III	57–59	CAR*^a^*	Ocular, respiratory
F	40, 41	III	51–52	CAR*^a^* (long fiber)	Enteric
G	52^†^	?	55	ND	Enteric

References are as follows;

a: [29,116],

b: [117],

c: [118],

d: [119],

e: [49,50],

f: [55],

g: [120–122].

* Type is the accepted term for Ad species which have been characterized by non-serological techniques.

† HAdV-B55, HAdV-D53, HAdV-D54 and HAdV-G52 were characterized using genomics and bioinformatics techniques and not by classical serum neutralization assays [9–11]. Abbreviations are as follows; CAR = coxsackie and adenovirus receptor, CD = cluster of differentiation, HSPG = heparan sulfate proteoglycan, ND = not determined.

**Table 2:** Current Clinical Studies in Humans Using Retargeted Adenoviral Vectors.

	**Type of Study**	**Virus Modification**	**Therapeutic/Imaging Transgene**	**Route of Delivery**	**Trial Location**	**References**
**Peptide Retargeting**	Phase I/II trial of Delta-24-RGD, a conditionally replicating adenovirus for recurrent malignant glioma	RGD peptide insertion in the HI loop of the fiber. 24 bp deletion in E1 region	No transgene	Intratumoral	MD Anderson Cancer Center, University of Texas, USA.	http://clinicaltrials.gov/
	Phase I trial of Ad5-D24RGD, a conditionally replicating adenovirus for ovarian and extraovarian cancer patients	RGD peptide insertion in the HI loop of the fiber. 24 bp deletion in E1 region	No transgene	Intraperitoneal	University of Alabama at Birmingham, Alabama, USA.	http://clinicaltrials.gov/
	Phase I study of Ad5.SSTR/TK.RGD for therapy and *in vivo* imaging in patients with recurrent ovarian and gynaecologic cancers	RGD peptide insertion in the HI loop of the fiber. 24 bp deletion in E1 region	Somatostatin receptor Herpes simplex virus thymidine kinase (HSV-TK)	Intraperitoneal	University of Alabama at Birmingham, Alabama, USA.	http://clinicaltrials.gov/ Matthews *et al*., 2009. [290].
	Phase I trial of conditionally replicating adenovirus (ICOVIR5) in patients with locally advanced or metastatic malignant melanoma	RGD peptide insertion, 24 bp deletion in E1A, E2F-modified promoter, insulator and Kozak sequence for E1A.	No transgene	Intravenous	Institut Catala d’Oncologia, Barcelona, Spain.	http://clinicaltrials.gov/ Cascallo *et al*., 2007. [291].
	Phase I/II trial of Delta-24-RGD, a conditionally replicating adenoviral vector, in patients with recurrent glioblastoma multiforme	RGD peptide insertion in fiber and 24 bp deletion in E1 region	No transgene	Convection enhanced delivery	VU University Medical Center Netherlands	http://clinicaltrials.gov/
	Compassionate clinical treatment of cancer patients with conditionally replicating adenovirus (ICOVIR7)	RGD peptide insertion, 24 bp deletion in E1A, enhanced E2F-modified promoter, insulator and Kozak sequence for E1A.	No transgene.	Intratumoral or intravenous	International Comprehensive Cancer Center Docrates and Eira Hospital, Helsinki, Finland.	Rojas *et al*., 2009; Nokisalmi *et al*., 2010. [292,294].
**Serotype Knob Pseudotyping**	Compassionate clinical treatment of cancer patients with fiber knob pseudotyped and conditionally replicating Ad5/3-Cox2L-D24	Ad3 knob substituted for Ad5, 24 bp deletion in E1A, substitution of E1A promoter with COX-2 promoter	No transgene	Intratumoral, Intravenous or intraperitoneal	International Comprehensive Cancer Center Docrates and Eira Hospital, Helsinki, Finland.	Pesonen *et al*., 2010. [295].

**Table 3:** Summary of Published Hexon Modifications for Adenovirus.

**Virus**	**Hexon Modification**	**Viability**	**Reference**
Av12LacZ	Substitution by Ad12 hexon gene	Poor growth	Roy *et al*., 1998. [331]
dlAd5NCAT-H2	Substitution by Ad2 hexon gene	Viable	Gall *et al*., 1998. [332]
dlAd5NCAT-H2 L2	Substitution by Ad2 (HVR7)	Viable	
Ad5-Ad7 hexon	Substitution by Ad7 (HVR7)	Non viable	
pAd5/Ad1 gag	Substitution by Ad1 hexon gene	Viable	Youil *et al*., 2002. [335]
pAd5/Ad6 gag	Substitution by Ad6 hexon gene	Viable	
pAd5/Ad7 gag	Substitution by Ad7 hexon gene	Non viable	
pAd5/Ad9 gag	Substitution by Ad9 hexon gene	Non viable	
pAd5/Ad10 gag	Substitution by Ad10 hexon gene	Non viable	
pAd5/Ad12 gag	Substitution by Ad12 hexon gene	Poor growth	
pAd5/Ad13 gag	Substitution by Ad13 hexon gene	Non viable	
pAd5/Ad15 gag	Substitution by Ad15 hexon gene	Non viable	
pAd5/Ad17gag	Substitution by Ad17 hexon gene	Non viable	
pAd5/Ad18 gag	Substitution by Ad18 hexon gene	Non viable	
pAd5/Ad19 gag	Substitution by Ad19 hexon gene	Non viable	
pAd5/Ad27 gag	Substitution by Ad27 hexon gene	Non viable	
pAd5/Ad35 gag	Substitution by Ad35 hexon gene	Non viable	
pAd5/Ad37 gag	Substitution by Ad37 hexon gene	Non viable	
Ad5BAP	BAP domain (71 aa) in HVR5 (aa 268–272)	Viable	Campos *et al*., 2004. [132]
Ad5/HVR2-His6	6His in HVR2 (aa 189–192)	Viable	Wu *et al*., 2005. [131]
Ad5/HVR3-His6	6His in HVR3 (aa 216–217)	Viable	
Ad5/HVR5-His6	6His in HVR5 (aa 271–279)	Viable	
Ad5/HVR6-His6	6His in HVR6 (aa 306–309)	Viable	
Ad5/HVR7a-His6	6His in HVR7 (aa 432–438)	Viable	
Ad5/HVR7b-His6	6His in HVR7 (aa 416–455)	Non viable	
Ad5HVR48(1)	Substitution by Ad48 (HVR1)	Viable	Roberts *et al*., 2006. [330]
Ad5HVR48(1–7)	Substitution by Ad48 (HVR1–7)	Viable	
AdHRGD	RGD motif insertion (11 aa) in HVR5 (aa 268–262)	Viable	Vigant *et al*., 2008. [64]
AdH(GA)8	HVR5 (aa 268-262) introducing G-A motif	Viable	
AdH(GA)24	HVR5 (aa 268-262) introducing G-A motif	Viable	
AdHAd2	HVR5 swap (aa 268-262) with corresponding amino acids from Ad2 (14 aa)	Viable	
AdHAd19	HVR5 swap (aa 268-262) with corresponding amino acids from Ad19 (17 aa)	Viable	
AdHAd30	HVR5 swap (aa 268-262) with corresponding amino acids from Ad30 (6 aa)	Viable	
Ad5CMVlacZ-HVR5(Ad26)	Ad26-HVR5	Viable	Alba *et al.*, 2009. [60]
Ad5CMVlacZ-HVR7(Ad26)	Ad26-HVR7	Viable	
Ad5CMVlacZ-HVR5+7(Ad26	Ad26-HVR5 and Ad26-HVR7	Viable	
Ad5CMVlacZ-HVR5*	HVR5 (T270P and E271G)	Viable	
Ad5CMVlacZ-HVR7*	HVR7 (I421G, T423N, E424S, L426Y)	Viable	
Ad5CMVlacZ-E451Q	HVR7-E451Q	Viable	
Ad5CMVlacZ-HVR5*7 *E451Q	HVR5 (T270P and E271G ) and HVR7(I421G,T423N,E424S, L426Y and E451Q)	Viable	
